# Predictive factors of the dimensions and location of mental foramen using cone beam computed tomography

**DOI:** 10.1371/journal.pone.0179704

**Published:** 2017-08-17

**Authors:** Juan Muinelo-Lorenzo, Ana Fernández-Alonso, Ernesto Smyth-Chamosa, Juan Antonio Suárez-Quintanilla, Jesús Varela-Mallou, María Mercedes Suárez-Cunqueiro

**Affiliations:** 1 Department of Surgery and Surgical Medical Specialties, Santiago de Compostela University, Santiago de Compostela, A Coruña, Spain; 2 Department of Psychiatry, Radiology and Public Health, Santiago de Compostela University, Santiago de Compostela, A Coruña, Spain; 3 Department of Morphological Sciences, Santiago de Compostela University, Santiago de Compostela, A Coruña, Spain; 4 Department of Organizational Psychology, Forensic Law, and Methodology of Behavioral Sciences, Santiago de Compostela University, Santiago de Compostela, A Coruña, Spain; 5 Department of Surgery and Medical Surgical Specialties, Medicine and Dentistry School, University of Santiago de Compostela, Spain, Health Research Institute of Santiago (IDIS), Santiago de Compostela, Spain; Medical University of South Carolina, UNITED STATES

## Abstract

**Objective:**

The mental foramen (MF) hosts main neurovascular structures, making it of crucial importance for surgical procedures. This study aimed to analyze the factors influencing the dimensions and location of the MF.

**Materials and methods:**

Cone beam computed tomography (CBCT) scans of 344 patients were examined for MF dimensions, as well as for the distances from the MF to the alveolar crest (MF-MSB), and to the inferior mandibular border (MF-MIB).

**Results:**

Gender, mandibular side and presence of accessory mental foramina (AMF) significantly influence MF area. Males, left hemimandibles, and hemimandibles with no AMF had a higher rate of large MF areas (B = − 0.60; p = 0.003, females; B = 0.55; p = 0.005; B = 0.85; p = 0.038). Age, gender and dental status significantly influence MF-MSB distance. The distance decreased as age increased (B = −0.054; p = 0.001), females showed a lower rate of long MF-MSB distances (B = −0.94, p = 0.001), and dentate patients showed a higher rate of long MF-MSB distances (B = 2.27; p = 0.001). Age, gender and emerging angle significantly influenced MF-MIB distance. The distance decreased as age and emerging angle increased (B = −0.01; p = **0**.001; B = −0.03; p = 0.001), and females had a lower rate of long MF-MIB distances (B = −1.94, p = 0.001).

**Conclusions:**

General and local factors influence the dimensions and location of MF. MF dimensions are influenced by gender, mandibular side, anteroposterior position, and the presence of AMF. Distance from MF to alveolar crest is influenced by gender, age and dental status, while the relative MF position is influenced by age and dental status. CBCT images make it possible to analyze the MF in order to avoid complications during surgical procedures.

## Introduction

The mental foramen (MF) is considered one of the main anatomic landmarks in the anterior region of the mandible because it constitutes the exit through which the terminal mandibular neurovascular branches pass. There are anatomical variations affecting the MF and mental nerve regarding location, size, emerging direction, number, and shape [1,2]. Since prehistoric times, the mandible of humans has undergone a decrease in its overall size compared to other primates [3,4]. The mental foramen (MF) has also varied its size and location. The MF has shifted from the molar region in Neolithic skulls to its current premolar position in modern humans. Moreover, the MF has increased in size since the Neolithic period [5].

The most common MF pattern of emergence in Caucasians and Maoris is the posterior direction, whereas right-angled emergence is predominant in Negro skulls [6]. There have been reports of subjects with double or multiple MFs [7]. The occurrence of these accessory mental foramina (AMF) has been explained by the early separation of the mental nerve prior to the complete formation of the MF [8], which does not occur until the twelfth week of intrauterine development [9].

The morphometric characteristics of MF are of special interest due to the clinical implications involved. MF can be injured during surgical procedures, resulting in paresthesia or anesthesia of the chin, lower lip, and gingiva from MF to midline [1,10]. It is critical for dental surgeons to accurately identify the MF prior to implant placement, orthognathic surgery, bone augmentation procedures and periapical surgery osteotomies [11–14]. An analysis of the factors having a possible influence on the size and position of the MF would allow for better understanding of MF variations prior to these procedures and help prevent vascular and nerve injuries. This study aimed to analyze the factors influencing MF dimensions and location.

## Material and methods

### Study population

The overall sample consisted of 357 patients for whom pre-operative CBCT imaging was performed from July 2008 to June 2014 for various clinical indications, in the Radiology Unit of the Medicine and Dentistry School at the University of Santiago de Compostela, Spain. Ethical approval for the study was obtained from the Galician Ethics Committee of Clinical Research (Ref. 2012/272). Written informed consent was obtained from all participants in the study.

The inclusion criteria were as follows: (1) whole mandibular body included in the CBCT volume; and (2) the CBCT voxel size was 0.3 mm or less. The exclusion criteria consisted of the following: (1) presence of any developmental or pathological conditions in the area of MF (i.e., tumors, cysts, or malformations); (2) presence of incompletely erupted teeth in the anatomic region; (3) evidence of mandibular fractures or surgical interventions, such as repositioning of inferior alveolar nerve or orthognatic surgery, and (4) presence of any artifacts or blurring affecting image quality.

### Image adquisition

CBCT exams were performed using i-CAT^®^ Model 17–19 (Imaging Sciences International Inc., Pensilvania, USA), with a flat panel detector of cesium iodure (CsI) made of amorphous silicon (a-Si). The following exposure settings were used: tube voltaje peak of 120 kVp, current of 5 mAs, and exposure time of 14.7 s. The patient's occlusal plane was set parallel to the floor.

Multiplanar CBCT reconstructions were jointly analyzed by two experienced researchers to identify MF and AMF. The projection data in DICOM files were reconstructed on computer (Samsung^®^ R522; Samsung Electronics, Seoul, South Korea) using Carestream^®^ CS 3D v.3.2.12 software (Carestream Health Inc., Rochester, NY, USA.). CBCT-reconstruction slice thickness was 0.3 mm.

### Measurement procedure

**Fig 1:**
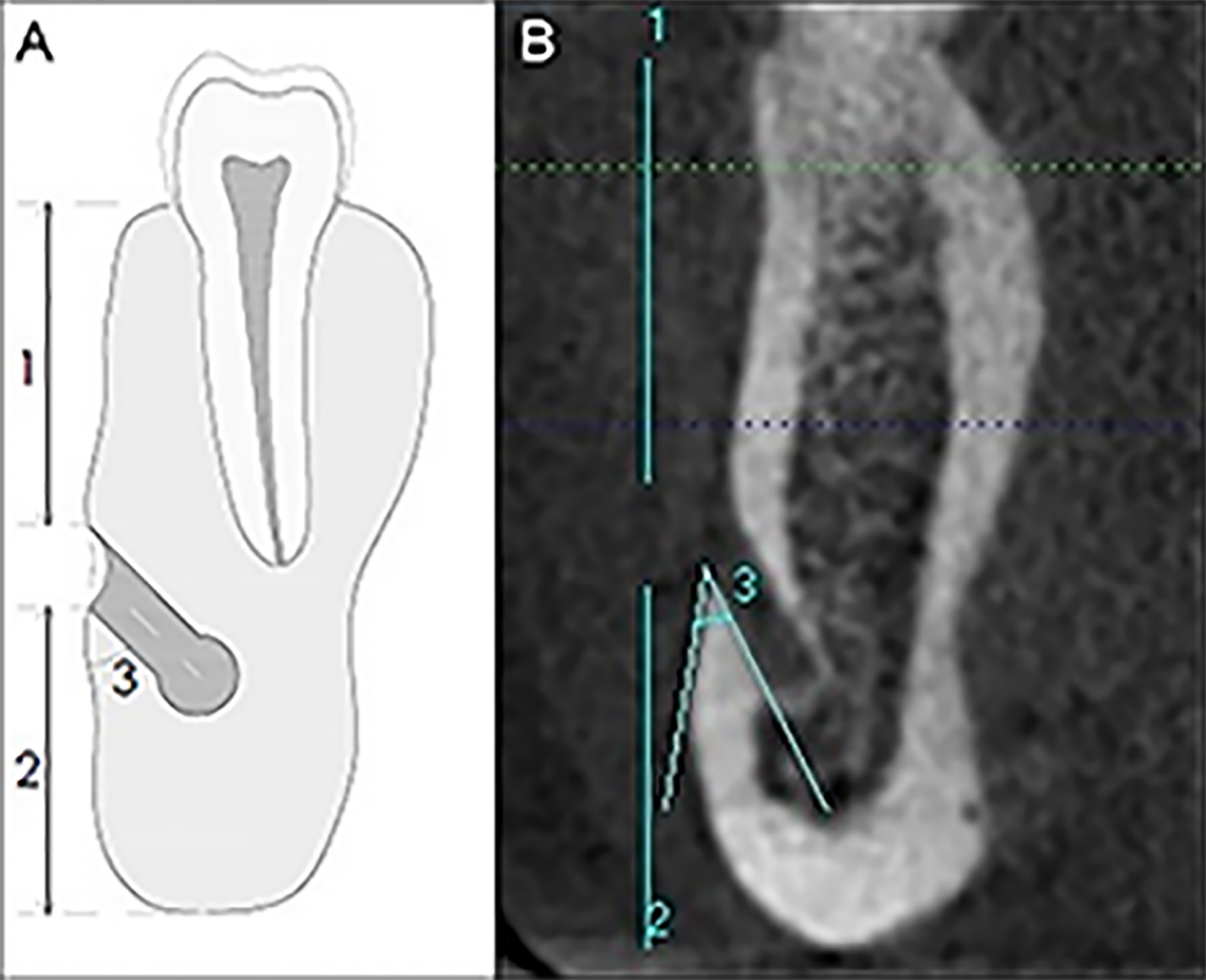
Location of the MF. (A) MF-MSB distance (1), MF-MIB distance (2), Emerging angle of the MF (3). (B) CBCT cross-sectional reconstructed slices of the mandible to show MF-MSB and MF-MIB distances, and emerging angle.

On cross-sectional slices, the position of MF in the mandibular body was determined by the distance from the alveolar bone crest to the MF superior border (MF-MSB), and the distance from the MF inferior border to the lower border of the mandible (MF-MIB). Mandibular vertical distance (MV) was calculated by the sum of the MF-MSB and MF-MIB distances. The crossing angle between the tangent to the vestibular mandibular surface and a parallel to the emerging direction of the mental nerve was considered the emerging angle (Fig 1). MF emergence was classified into four types according to its exit direction: I) superior, II) posterior, III) direct, and IV) anterior. The anteroposterior location of MF was classified with respect to mandibular teeth from the 1st premolar to 1st molar. Dental status was classified into three groups: dentate (6 or more remaining teeth), partially dentate (less than 6 remaining teeth) and edentulous.

**Fig 2:**
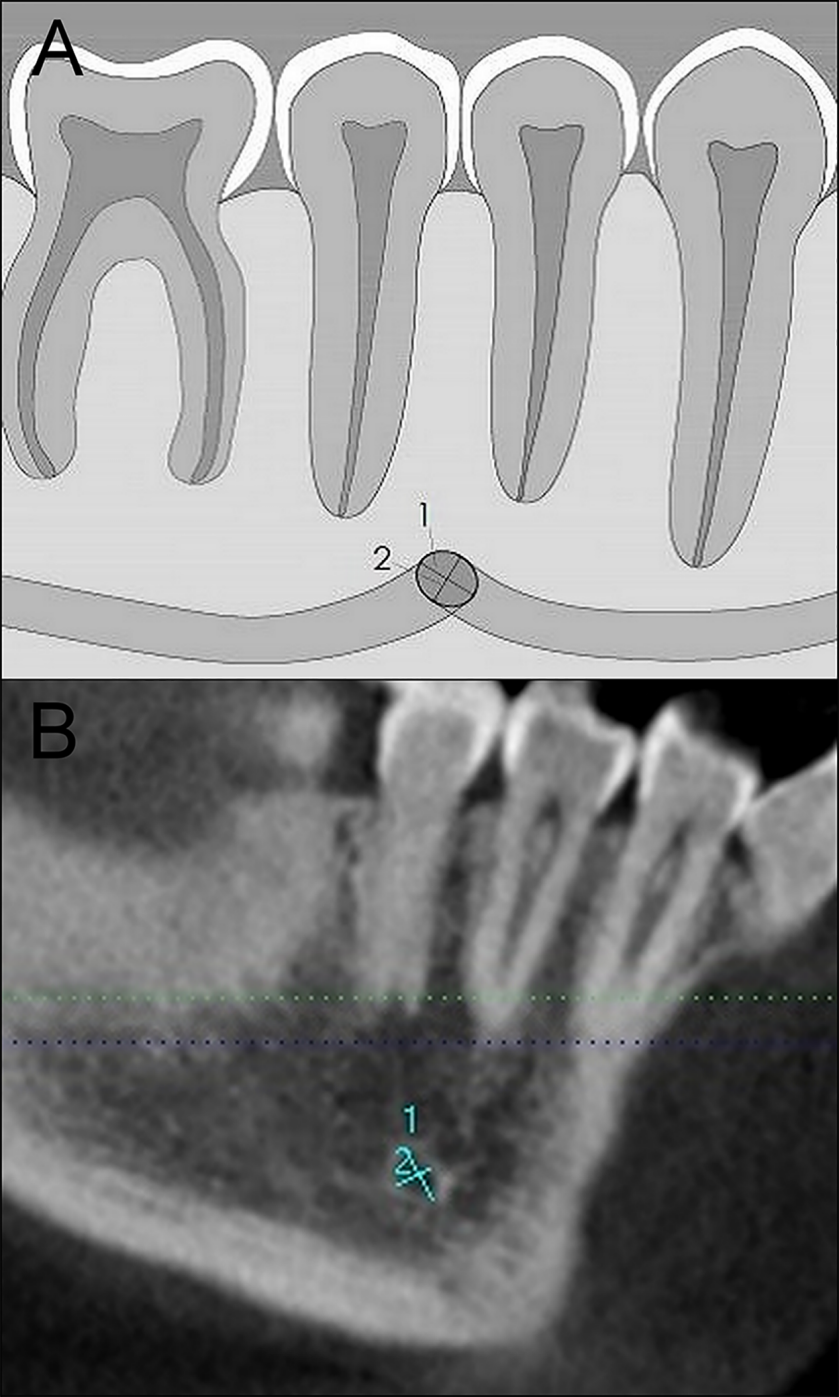
Dimensions of the MF. (A) Long diameter (1), Short diameter (2). (B) CBCT sagittal reconstructed slices of the mandible to show the long and short diameters of the MF.

Long and short axes of each MF were measured on CBCT sagittal reconstructions; in addition, MF areas were calculated using the formula: oval area, A = *abπ*/4; [where *a* = long diameter and *b* = short diameter] (Fig 2). An AMF was defined as an ancillary buccal foramen presenting continuity with the mandibular canal. AMF had a smaller diameter than MFs. CBCT measurements were carried out by an experienced researcher under standard conditions (a 15.6 inch monitor in a dimly lit room). Intra-observer variability was analyzed by re-measuring 40 randomly selected CBCT images 1 month later.

### Statistical analysis

Statistical analysis was performed using SPSS^®^ (v. 21.0, IBM Corporation, NY, USA). Descriptive data included mean ± standard deviation (SD). Intra-observer and inter-observer agreement were assessed using the intraclass correlation coefficient. The t-test was used for paired comparisons in MF morphometric characteristics between gender and age groups. The one-way ANOVA and post hoc Bonferroni multiple comparison tests were used to compare MF characteristics in relation to dental status and MF position. A polar group strategy was used to obtain dichotomized variables. For each dependent variable two groups were established: the first group was ≤33^rd^ percentile, and the second group was ≥66^th^ percentile. A binary logistic regression analysis was performed to determine the factors influencing MF dimensions and location. Regression analysis was further performed to analyze the influence of age on MF-MSB distance in fully dentate patients. Statistical significance was set at p < 0.05.

## Results

The final sample consisted of 344 CBCT scans selected from the initial 355. Three CBCTs were excluded from the initial sample due to partially erupted or included teeth in the region of interest, six scans presented pathology (four patients had radiolucident areas consistent with granulomas or cysts, one patient had lesions consistent with cherubism, and another had multiple dental inclusions and lesions suggesting cysts), and two CBCTs did not have adequate quality for analysis. The population consisted of 205 females (59.6%) and 139 males (40.4%) (mean age 47.44 ± 15.52 years; range: 13–86).

Of the total 688 MFs, the mean long diameter was 4.44 ± 1.13 mm, the mean short diameter was 2.92 ± 0.75 mm, and the mean area was 10.62 ± 5.00 mm^2^. MF short diameter and MF area were significantly higher on the left side (p = 0.000, p = 0.013). The MF-MIB distance was 13.55 ± 1.06 mm, and MF-MSB distance was 11.42 ± 3.34 mm. The mean MF emerging angle from the mandible was 53.45 ± 15.90°. Male subjects presented statistically higher MF dimensions (long diameter, short diameter, and area) (p = 0.000, p = 0.000, p = 0.000, respectively), distances (MF-MIB and MF-MSB) (p = 0.000, p = 0.000, respectively), and MF emerging angle (p = 0.028). Younger subjects presented statistically higher MF-MSB distance (p = 0.000), while older subjects presented statistically higher MF emerging angle (p = 0.000). Differences were also found with respect to dental status. Dentate subjects had significantly higher MF dimensions (long diameter and area) than edentulous patients (p = 0.013, p = 0.038). Dentate patients also had statistically higher MF-MSB distance (p = 0.000), while MF emerging angle was statistically higher in edentulous patients (p = 0.000) (Table 1).

**Table 1:** MF morphometric characteristics with respect to dental status.

	Dentate	Partially dentate	Edentulous	
MF CHARACTERISTICS	(n = 614)	(n = 28)	(n = 46)	*P*
*Long Diameter*	2.93 ± 0.76	2.96 ± 0.60	2.67 ± 0.79	0.075
*Short Diameter*	4.47 ± 1.12*	4.42 ± 1.09	3.97 ± 1.16*	**0.013**
*Area*	10.71 ± 5.07*	10.57 ± 4.15	8.75 ± 4.56*	**0.038**
*MF-MIB*	13.52 ± 1.59	13.47 ± 1.79	13.89 ± 1.63	0.325
*MF-MSB*	11.84 ± 3.02*	9.68 ± 3.13†	6.77 ± 3.75*†	**0.000**
*Emerging Angle*	52.07 ± 14.68*	54.54 ± 18.35†	71.20 ± 19.33*†	**0.000**

MF characteristics are expressed as means ± standard deviation. The ANOVA was performed to compare MF characteristics with respect to dental status. Results in bold letters indicate statistical significance (p<0.05).

*, and † indicates a significant difference after Bonferroni post-hoc multiple tests.

MF, mental foramen; MF-MIB, distance from the alveolar crest to the MF; MF-MIB, distance from the MF to the inferior mandibular border.

**Fig 3:**
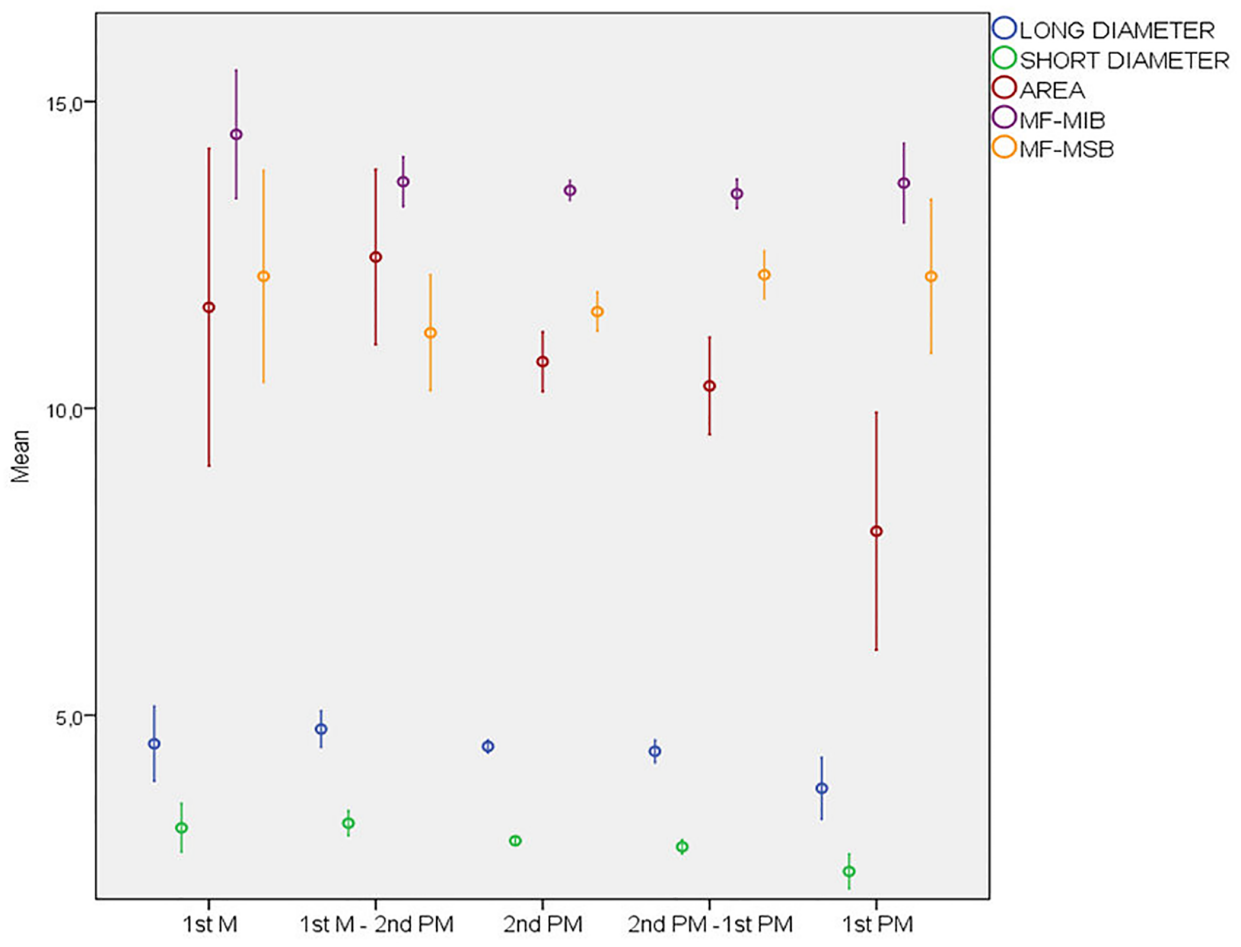
MF morphometric characteristics with respect to anteroposterior position. MFs presented a statistically significant reduction in dimensions (long diameter, short diameter, and area) as their position moved closer to the midline. The area and long diameter of the MFs located below the 2nd premolar and those located between 1st molar and 2nd premolar were significantly higher compared to the MFs located below 1st premolar. The short diameter of the MFs located below the 1st molar, between 1st molar and 2nd premolar, and below the 2nd premolar was significantly higher compared to the MFs located below the 1st premolar. In addition, the short diameter of the MFs located between the 1^st^ molar and 2^nd^ premolar was significantly higher compared to the MFs located between the 2^nd^ premolar and 1^st^ premolar.

The anteroposterior location of MF in relation to mandibular teeth presented the following distribution: 2.7% were below the 1^st^ molar, 9.1% between the 1^st^ and 2^nd^ molars, 57.9% below the 2^nd^ premolar, 25.3% between the 2^nd^ and 1^st^ premolar, and 5.0% were below the 1^st^ premolar. MFs presented a statistically significant reduction in dimension (long diameter, short diameter, and area) as their position moved closer to the midline (Table 2) (Fig 3).

**Table 2:** MF morphometric characteristics with respect to anteroposterior location.

	1^st^ M	1^st^ M-2^nd^ PM	2^nd^ PM	2^nd^ PM-1^st^ PM	1^st^ PM	
CHARACTERISTICS	(n = 17)	(n = 58)	(n = 370)	(n = 161)	(n = 32)	*P*
*Long Diameter*	4.54 ± 1.19	4.77 ± 1.14*	4.49 ± 1.03†	4.41 ± 1.20	3.81 ± 1.40*†	**0.003**
*Short Diameter*	3.17 ± 0.78*	3.24 ± 0.79†¶	2.96 ± 0.72∫	2.85 ± 0.77†	2.45 ± 0.79*¶∫	**0.000**
*Area*	11.64 ± 5.03	12.46 ± 5.44*	10.75 ± 4.77†	10.36 ± 5.11	7.99 ± 5.37*†	**0.001**
*MF-MIB*	14.47 ± 2.04	13.69 ± 1.54	13.55 ± 1.60	13.50 ± 1.55	13.67 ± 1.80	0.194
*MF-MSB*	12.15 ± 3.36	11.23 ± 3.60	11.58 ± 3.12	12.18 ± 2.53	12.15 ± 3.49	0.144
*Emerging Angle*	47.12 ± 12.04	55.22 ± 16.28	52.21 ± 15.13	52.73 ± 14.03	52.66 ± 15.77	0.365

MF characteristics are expressed as means ± standard deviation. The ANOVA was performed to compare MF characteristics with respect to MF anteroposterior location. Results in bold letters indicate statistical significance (p < 0.05).

*, †, ¶, and ∫ indicates a significant difference after Bonferroni post-hoc multiple tests.

M, molar; PM, premolar; MF, mental foramen; MF-MIB, distance from the alveolar crest to the MF; MF-MIB, distance from the MF to the inferior mandibular border.

The predominant type of MF emergence was superior direction, followed in frequency by direct, posterior and anterior types. No statistically significant differences were observed with regard to gender (p > 0.05). However, there were statistical differences between age groups (p = 0.000). Patients under 50 years of age had superior emergence MFs more frequently than older patients. Conversely, patients over 50 had a higher number of direct-type MFs. Regarding the anteroposterior position, no statistically significant differences were found in emergence type. However, there were statistically significant differences according to dental status (p = 0.000). Dentate patients had mostly superior-direction MFs, while partially dentate and edentulous patients had mostly direct emergence.

MF morphometric characteristics varied significantly depending on the type of emergence. Statistically significant differences in long diameter, MF-MSB and MF-MIB distances were observed. The MF-MIB distance was statistically higher in superior-type MFs as compared to direct-emergence MFs (p = 0.034). In addition, MF-MSB distance was statistically higher in superior-type MFs compared to direct-emergence MFs and anterior-emergence MFs (p = 0.005, p = 0.000). Furthermore, this distance was statistically higher in posterior-emergence MFs than anterior-emergence MFs (p = 0.042) (S1 Dataset). Inter-observer variability was an intraclass correlation mean value of 0.748, ranging from 0.62 to 0.85. Intra-observer variability was an intraclass correlation mean value of 0.799, ranging from 0.61 to 0.91 (S2 Dataset).

### Predicting the dimensions of the mental foramen

The regression results indicated that gender, mandibular side, anteroposterior MF position and AMF presence significantly influence MF short diameter (p < 0.05). Females had a significantly lower rate of large MF short diameters (B = −0.73; p = 0.001). Regarding mandibular side, there was a higher rate of large MF short diameters on the left side (B = 0.71; p = 0.001). In relation to the anteroposterior position, the MFs situated below the 1^st^ molar (B = 1.85; p = 0.026), between the 1^st^ molar and 2^nd^ premolar (B = 2.03; p = 0.001), and below the 2^nd^ premolar (B = 1.03; p = 0.038) had a higher rate of large MF short diameters in comparison to the MFs situated below the 1^st^ premolar. With respect to those located below the 1^st^ premolar, the rate of large MF short diameters was 6.3 times greater below the 1^st^ molar, 7.6 times greater between the 1^st^ molar and 2^nd^ premolar, 2.8 times greater below the 2^nd^ premolar, and 2.7 times greater between the 2^nd^ and 1^st^ premolar (Table 2) (Fig 3).

In addition, hemimandibles with no AMF showed a higher rate of large MF short diameters (B = 0.90; p = 0.038). The rate of large MF short diameters was nearly 2.5 times higher in hemimandibles with no AMF presence. The regression analysis showed that gender and presence of AMF had a significant influence on MF long diameter (p < 0.05).

**Fig 4:**
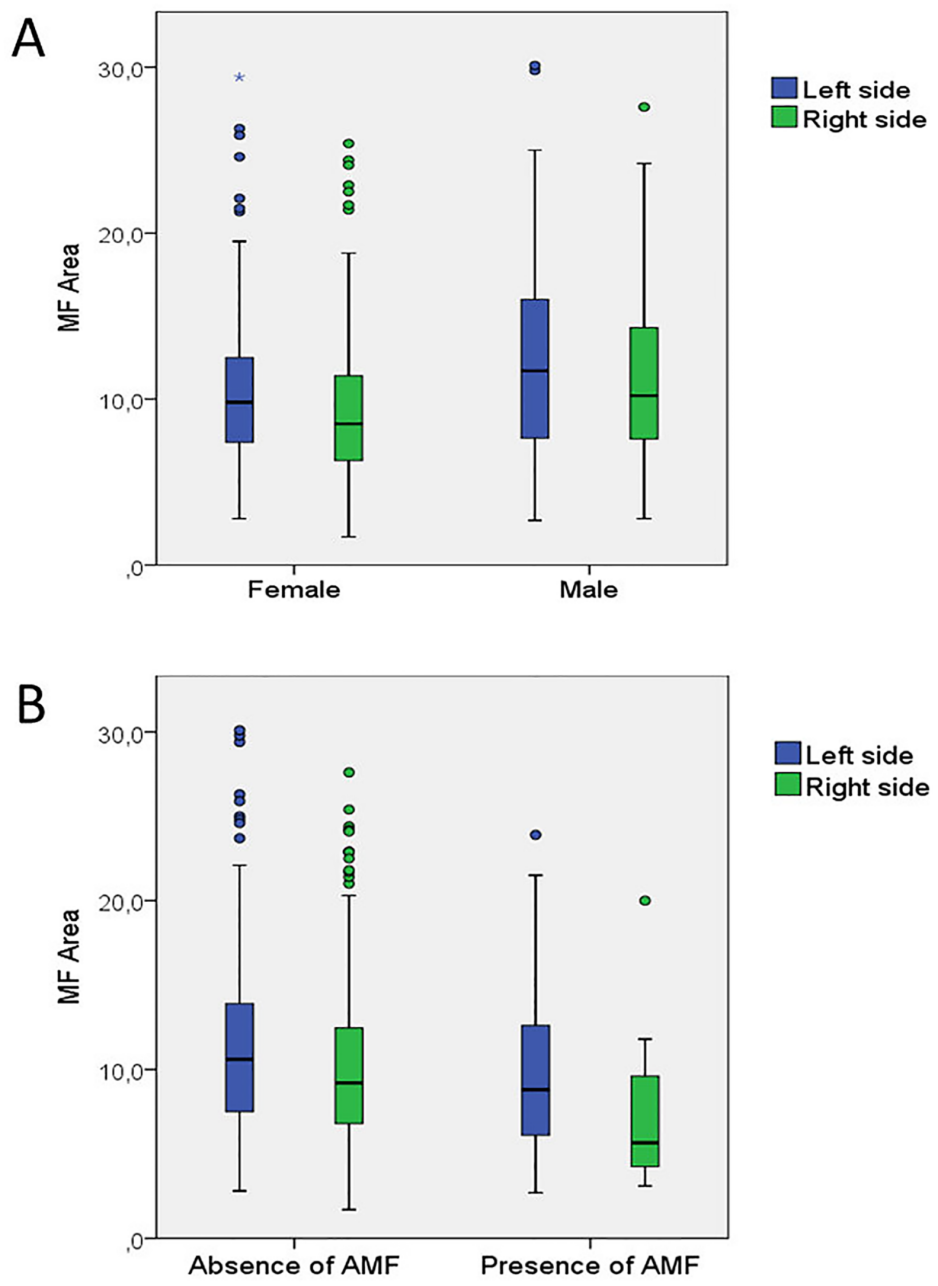
Distribution of MF area. (A) Females had a lower rate of large MF areas (B = −0.60; p < 0.01). Left hemimandibles had a higher rate of large MF areas (B = 0.55; p < 0.01). (B) Hemimandibles with no AMF had a higher rate of large MF areas (B = 0.85; p < 0.05).

The regression analysis showed that gender, mandibular side and the presence of AMF significantly influence MF area (p < 0.05). Females had a lower rate of large MF areas (B = −0.60; p = 0.003). The likelihood of presenting a large MF area was 1.8 times greater in males (1/0.54; OR in females = 0.54). Regarding side, left hemimandibles had a higher rate of large MF areas (B = 0.55; p = 0.005). The likelihood of having large MF area was 1.7 times greater in left hemimandibles. In addition, hemimandibles with no AMF had a higher rate of large MF areas (B = 0.85; p = 0.038). The likelihood of having large MF area was 2.3 times greater in hemimandibles with no AMF (Table 3) (Fig 4), (S3 Dataset).

**Table 3:** Determining factors of MF long diameter, short diameter and area.

	B	P	OR	(95%CI)
***Long Diameter***				
*Gender (female)*	−0.50	**0.01**	0.60	(0.41,0.88)
*Presence of AMF*	−0.80	**0.04**	0.23	(1.02,4.84)
***Short Diameter***				
*Gender (female)*	−0.73	**0.001**	0.47	(0.31,0.72)
*Mandibular side (left)ft)*	0.71	**0.001**	2.05	(1.34,3.12)
*Anteroposterior position of MF*				
*1st M*	1.85	**0.026**	6.35	(1.24,32.51)
*1st M-2nd PM*	2.03	**0.001**	7.61	(2.26,25.65)
*2nd PM*	1.03	**0.038**	2.82	(1.06,7.53)
*Absence of AMF*	0.90	**0.038**	2.46	(1.05,5.76)
***Area***				
*Gender (female)*	−0.60	**0.003**	0,54	(1.00,1.03)
*Mandibular side (left)*	0.55	**0.005**	1.74	(0.09,0.22)
*Presence of AMF*	0.85	**0.038**	2.34	(0.95,0.98)

Binary logistic regression to determine the general and local factors influencing MF dimensions: Results in bold letters indicate statistical significance (p < 0.05). MF, mental foramen; AMF, accessory mental foramen; M, molar; PM, premolar; OR, odds ratio.

### Predicting the location of the mental foramen

The binary logistic regression showed that age, gender and dental status significantly influence MF-MSB distance (p < 0.05). This distance decreased as age increased (B = −0.054; p = 0.001). In terms of gender, females had a lower rate of long MF-MSB distances (B = −0.94, p = 0.001). The likelihood of having a long MF-MSB distance was 2.56 times higher in males (1/0.39; OR in females = 0.39). In addition, dentate patients showed a 10-times higher rate of long MF-MSB distances in comparison with edentulous patients (B = 2.27; p = 0.001).

**Fig 5:**
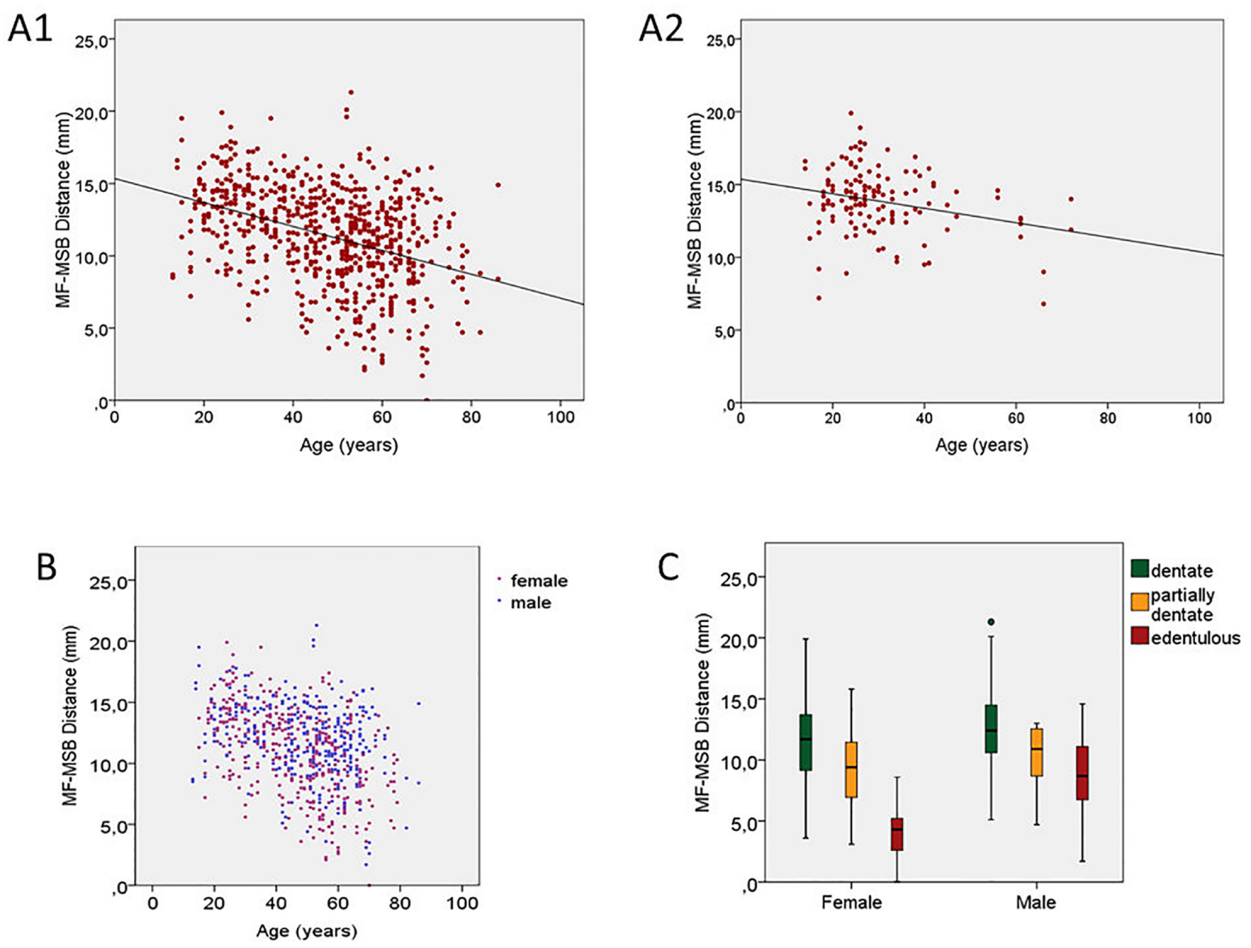
Progress of MF-MSB distance. (A1) Progress in total population over time. (A2) Progress in completely dentate patients over time (R = 0.26; B = −0.05; p = 0.02) †. (B) Females showed a lower rate of large MF areas (B = −0.60; p < 0.01) *. (C) Dentate patients showed a higher rate of long MF-MSB distances in comparison with edentulous patients (B = 2.27; p < 0.01) *. *Binary logistic regression analysis. †Linear regression analysis.

Regarding completely dentate patients, the linear regression analysis revealed that age had an influence on MF-MSB distance (R = 0.26; B = −0.05; p = 0.02) and MF-MSB/MV ratio (R = 0.32; B = −0.001; p < 0.01) (Fig 5).

The binary logistic regression showed that age, gender and emerging angle significantly influenced the MF-MIB distance (p < 0.05). The distance decreased as age increased (B = −0.01; p = 0.001). In terms of gender, females had a lower rate of long MF-MIB distances (B = −1.94, p = 0.001). The likelihood of having a long MF-MIB distance was 7.14 times higher in males (1/0.14; OR in females = 0.14). In addition, as emerging angle increased, the likelihood of presenting long MF-MIB distances decreased (B = −0.03; p = 0.001).

The binary logistic regression showed that age, gender, dental status and emerging angle significantly influenced the MV distance (p < 0.05). This distance decreased as age increased (B = −0.04; p = 0.000). In terms of gender, females had a lower rate of long MV distances (B = −1.96, p = 0.000). The likelihood of having a long MV distance was 7.14 times higher in males (1/0.14; OR in females = 0.14). In addition, dentate patients showed an almost 5 times higher rate of long MV distances as compared to edentulous patients (B = 1.59; p = 0.001). Regarding emerging angle, the higher the angle the lower the likelihood of presenting long MV distances (B = −0.03; p = 0.000).

The binary logistic regression showed that age and dental status significantly influenced the MF-MSB/MV ratio (p < 0.05). The ratio decreased as age increased (B = −0.05; p = 0.000). In addition, dentate patients showed a 10 times higher rate of long MF-MSB/MV ratios in comparison to edentulous patients (B = 2.37; p = 0.001) (Table 4), (S4 Dataset).

**Table 4:** Determining factors of MF-MSB distance, MF-MIB distance, MV distance and MF-MSB/MV ratio.

	B	P	OR	(95%CI)
***MF-MSB distance***				
*Age*	−0.05	**0.001**	0.95	(0.93,0.96)
*Gender (female)*	−0.94	**0.001**	0.39	(0.25,0.61)
*Dental status*				
Dentate	2.27	**0.001**	9.67	(2.82,3.18)
Partially dentate	1.10	0.19	3.01	(0.57,15.96)
***MF-MIB distance***				
*Age*	−0.01	**0.01**	1.01	(1.00,1.03)
*Gender (female)*	−1.94	**0.001**	0.14	(0.25,0.22)
*Emerging angle*	−0.03	**0.001**	0.97	(0.95,0.98)
**MV distance**				
Age	−0.04	**0.000**	0.95	(0.94,0.97)
Gender (female)	−1.96	**0.000**	0.14	(0.08,0.22)
Dental status				
Dentate	1.59	**0.001**	4.93	(2.82,3.18)
Partially dentate	1.03	0.164	2.81	(0.65,12.09)
Emerging angle	−0.03	**0.000**	0.97	(0.95,0.98)
**MF-MSB/MV Ratio**				
Age	−0.05	**0.000**	0.92	0.94
Dental Status				
Dentate	2.37	**0.001**	10.78	(2.52,46.05)
Partially dentate	1.69	0.071	5.45	(0.86,34.35)

Binary logistic regression to determine the general and local factors influencing MF location: Results in bold letters indicate statistical significance (p < 0.05). MF, mental foramen; AMF, accessory mental foramen; MF-MIB, distance from the alveolar crest to the MF; MF-MIB, distance from the MF to the inferior mandibular border; MV: mandibular vertical distance (MF-MSB + MF-MIB) M, molar; PM, premolar; OR, odds ratio.

## Discussion

This study has identified a number of factors influencing MF size. Gender, mandibular side, MF anteroposterior position, and the presence of AMF largely determine variations in MF area and MF diameter. Although there are several studies analyzing MF size [15,16], to the best of our knowledge, this is the first study to evaluate whether MF size is influenced by local and general factors using regression model analysis. Previous studies have carried out only a comparative study by age and gender [16–19]. Regarding general factors, in a CT study Fujita et al. [20] reported no differences in MF dimensions between males and females. Using a different methodological approach, Chen et al. [21] considered the bucco-lingual diameter of the intraalveolar canal at the side of MF to be similar to MF diameter. Mean diameters in their study were 2.26 mm in the American population and 2.13 mm in Taiwanese population. They found no significant differences in MF dimensions with respect to gender or age.

In contrast, other CBCT studies and studies on dry mandibles reported that males had significantly greater dimensions [10,17–19], which is consistent with the present study. Particularly, Kalender et al. [18] observed greater dimensions among males in both MF height and width. Other authors found only differences in height [17,19], or only in width [10]. In our regression model, gender was found to be a determining factor in MF size: males were twice as likely to have large MFs (>66th percentile). Age is another independent variable with a possible relation to MF dimensions. Orhan et al. [22] described that children aged 6 to 12 years had significantly smaller MF dimensions compared to older children. However, like other adult studies [17,19], we found that age had no influence on MF dimensions.

Regarding local factors, we found both MF diameter and MF area were significantly influenced by mandibular side, MF being greater in left hemimandibles. Philips et al. [23] described similar findings, but this contrasts with other CBCT and CT research [18,19,24], which report no variation in MF dimensions according to mandibular side. In addition, MF dimensions change according to anteroposterior position. MF diameter decreases as its position becomes more anterior in the mandible. This seems more the result of craniocaudal reduction than of mandibular canal branching. A similar decrease has also been observed in other mandibular vascular structures such as the incisive canal [25,26].

In a previous study involving 344 patients with a total of 48 AMF [27], the present research team found that MF dimensions were smaller in the presence of AMF. There are only four studies by other authors analyzing mental foramen dimensions with respect to the presence of accessory mental foramina [28–31]. These authors found that MF dimensions were smaller in hemimandibles with AMF, but unlike our previous study, they reported no significant differences [28,30], which may be due to the small sample size. Furthermore, in our regression model the presence of AMF is a determining factor for MF dimensions. This could be due to the fact that some parts of the neurovascular bundles that pass through the MF are transferred to the AMF, thus, MF dimensions become smaller [32]. To date, no previous study has performed a regression analysis to assess the influence of these factors. Moreover, this is the first regression study to include the presence of AMF as one of its factors.

The present study has also identified a number of factors influencing the distance from MF to the alveolar crest and from MF to the mandibular lower border. Age, gender, emerging angle, and dental condition are associated with variations in these distances. In addition, age and dental condition have also been identified as influencing factors in MF vertical position. The location of MF varies throughout life. Before tooth eruption, children present MFs closer to the alveolar crest. During eruption, the location descends to an intermediate position between alveolar crest and inferior mandibular border; and in dentate adults, it is closer to the inferior mandibular border [33]. In this sense, Orhan et al. [22] described that the distance from MF to the alveolar crest varies significantly among children aged 6–12, children aged 13–15, and children aged 16–18 years; presenting lower distances in the youngest group. This apparent movement of MF position is relative and depends on alveolar bone apposition [15]. However, we must be aware that there are several factors that may affect MF position such as periodontal disease, postextraction bone loss, and bone loss by trauma [33]. MF-MIB and MF-MSB distances in the present study were consistent with previous research [10,19,34,35]. It has been shown that MF location is between 12 to 15.5 mm above the MIB [16,34,36–43]. Like other studies [18,19,37,39], we found no significant variations in these distances with respect to hemimandible side. With respect to gender, females showed significant lower MF-MSB and MF-MIB distances, in line with previous research [10,18,19,21,34,37,44]. In addition, in the present study the regression model showed that gender is a determining factor for MF-MSB and MF-MIB distances, as well as for MV distance. Despite the fact that MF-MSB, MF-MIB and MV distances were higher in males, we did not find gender to represent a determining factor in the vertical position of MF in the mandible. These gender differences have been described by Apinhasmit et al. [10], who also observed that the vertical MF position was not influenced by gender.

Regarding age, MF-MSB distance is shorter in the older age group, perhaps due to bone resorption resulting from tooth loss, which is more prevalent with increasing age. Similar differences have been observed by some authors [15,33,45,46]; however, others [19,47,48], found no influence of age on MF-MSB distance in patients presenting all teeth in the MF region. They claim that the position of the mandibular canal and the MF remain relatively constant regardless of age and gender [47,48]. However, our linear regression revealed that in fully dentate patients, age influences MF-MSB distance and MF vertical position (MF-MSB/MV ratio). Thus, as age increased, shorter MF-MSB distance and MF-MSB/MV ratio were observed. One of the factors that may explain these results is periodontal bone loss. Further research is needed to determine whether more factors are involved.

Regarding dental condition, we observed that tooth loss results in a decrease in MF-MSB distance, which is in line with the literature [15,33,38]. Soikkonen et al. [49] reported that it was 3.8 mm lower in edentulous than in dentate mandibles. Chu et al. [38] reported this occurring more acutely in males. The present study found that dental status is a predictive factor for MF-MSB distance. Thus, the likelihood of long MF-MSB distances is 10 times higher in dentate patients than in edentulous patients. On the other hand, unlike previous research [34,38,50], we found no variation in the MF-MIB distance with respect to dental status. Chrcanovic et al. [34] reported a reduction in MV in edentulous patients. These authors explain that dental absence results in greater changes in mandibular height than in mandibular width dimensions, and that dental status has a greater influence on the mandibular anatomy than does gender. Moreover, they describe that the relative MF position in the mandible presents statistically significant differences between dentate and edentulous patients. Following dental extraction, the MF is located closer to the alveolar crest. Our findings showed that dental status is an influencing factor of vertical MF position (MF-MSB/MV ratio). Regarding anteroposterior MF position, unlike other authors [19], who found an increase in the MF-MSB distance as MF position becomes more anterior, we found that MF-MSB distance does not vary significantly according to its anteroposterior position. This may be because that in our study not all patients were fully dentate in the MF region. of the bone resorption in the MF region in patients who are not fully dentate. The MF contains critical neurovascular structures, and represents a mandibular landmark with great importance for surgery. An injury of the MF as a consequence of inadequate planning may cause sensory disturbances for up to 6–16 months postsurgery. This has an incidence of 8.5% to 24% [1,12]. To prevent these complications, an accurate knowledge on the inferior alveolar nerve, mental nerve and MF anatomy is crucial. This study provides information regarding MF location that is vital to attaining mental nerve blockage.

## Conclusions

Based on the present study, MF dimensions are associated with gender, mandibular side, anteroposterior position, and the presence of AMF. Location of the MF is associated to general influencing factors such as gender, and patient age. It is also associated with local influencing factors such as dental status and emerging angle. CBCT images make it possible to detect and analyze the anatomical characteristics of MF in order to avoid complications associated to surgical procedures. Future studies involving different ethnic populations and larger population samples should be carried out to confirm these results.

## Supporting information

S1 Dataset Statistical analysis including mental foramen descriptive data, paired comparisons t-tests, and one-way ANOVA. (DOCX)

S2 Dataset Interobserver and intraobserver variability. (DOCX)

S3 Dataset Prediction of the mental foramen dimensions. (DOCX)

S4 Dataset Prediction of the mental foramen location. (DOCX)
