# Supplementary material for: Predictive factors of the dimensions and location of mental foramen using cone beam computed tomography
Source: PLoS One. 2017 Aug 17;12(8):e0179704. doi: 10.1371/journal.pone.0179704 (PMC5560523; doi:10.1371/journal.pone.0179704)
Supplement: S1 Dataset — (DOCX) [file pone.0179704.s001.docx]

**1.- MF CHARACTERISTICS**

| **Descriptive statistics** | | | | | |
| --- | --- | --- | --- | --- | --- |
|  | N | Mínimun | Máximum | Mean | *Sd* |
| MF EMERGING ANGLE | 688 | 26 | 135 | 53,45 | 15,906 |
| MF LONG DIAMETER | 688 | 1,9 | 8,7 | 4,441 | 1,1319 |
| MF SHORT DIAMETER | 688 | 1,1 | 6,5 | 2,922 | ,7595 |
| MF AREA | 688 | 1,7 | 40,3 | 10,600 | 5,0115 |
| MF-MIB | 688 | 9,5 | 18,8 | 13,550 | 1,6054 |
| MF-MSB | 688 | ,0 | 21,3 | 11,416 | 3,3463 |
| N valid (according to list) | 688 |  |  |  |  |

**MF ANTEROPOSTERIOR POSITION**

| **FM POSITION** | | | | | |
| --- | --- | --- | --- | --- | --- |
|  | | Frequency | Percentage | Valid percentage | Accumulated percentage |
| Valid | 1st MOLAR | 17 | 2,5 | 2,7 | 2,7 |
|  | 1st MOLAR-2nd PREMOLAR | 58 | 8,4 | 9,1 | 11,8 |
|  | 2nd PREMOLAR | 369 | 53,6 | 57,8 | 69,6 |
|  | 2nd PREMOLAR-1st PREMOLAR | 162 | 23,5 | 25,4 | 95,0 |
|  | 1ºPREMOLAR | 32 | 4,7 | 5,0 | 100,0 |
|  | Total | 638 | 92,7 | 100,0 |  |
| Lost | System | 50 | 7,3 |  |  |
| Total | | 688 | 100,0 |  |  |

**2. MF CHARACTERISTICS BY MANDIBULAR SIDE**

| **Group statistics** | | | | | | | | | | | | | |  |
| --- | --- | --- | --- | --- | --- | --- | --- | --- | --- | --- | --- | --- | --- | --- |
|  | | AGE GROUPS | | N | | MEAN | | SD | | Standart error of the mean | | | |  |
| FM EMERGING ANGLE | | Right | | 344 | | 53,36 | | 15,565 | | ,0839 | | | |  |
|  |  | Left | | 344 | | 53,54 | | 16,626 | | ,0877 | | | |  |
| FM LONG DIAMETER | | Right | | 344 | | 4,379 | | 1,1546 | | ,0606 | | | |  |
|  |  | Left | | 344 | | 4,502 | | 1,1091 | | ,0614 | | | |  |
| FM SHORT DIAMETER | | Right | | 344 | | 2,817 | | ,7594 | | ,0409 | | | |  |
|  |  | Left | | 344 | | 3,026 | | ,7462 | | ,0402 | | | |  |
| FM AREA | | Right | | 344 | | 10,107 | | 4,9958 | | ,2694 | | | |  |
|  |  | Left | | 344 | | 11,053 | | 5,0110 | | ,2702 | | | |  |
| MF-MIB | | Right | | 344 | | 13,576 | | 1,6082 | | ,0867 | | | |  |
|  |  | Left | | 344 | | 13,524 | | 1,6046 | | ,0865 | | | |  |
| MF-MSB | | Right | | 344 | | 11,253 | | 3,3176 | | ,1789 | | | |  |
|  |  | Left | | 344 | | 11,579 | | 3,3717 | | ,1818 | | | |  |
| **Independent simple T-test** | | | | | | | | | | | | | | |
|  | | | Levene test for equality of variances | | T test for the equality of averages | | | | | | | | | |
|  |  |  | F | Sig. | t | | df | Sig. (bilateral) | Mean difference | | Standart error of the difference | 95% confidence interval for the difference | | |
|  |  |  |  |  |  |  |  |  |  |  |  | Inferior | Superior | |
| EMERGING  ANGLE | Equal variances have been assumed | | ,719 | ,397 | -,144 | | 686 | ,896 | -,9460 | | ,3815 | -1,6950 | -,1969 | |
|  | Equal variances have not been assumed | |  |  | -,144 | | 684,688141 | ,896 | -,9460 | | ,3815 | -1,6950 | -,1969 | |
| LONG  DIAMETER | Equal variances have been assumed | | ,094 | ,759 | -1,433 | | 686 | ,152 | -,1235 | | ,0862 | -,2929 | ,0458 | |
|  | Equal variances have not been assumed | |  |  | -1,433 | | 685,896 | ,152 | -,1235 | | ,0862 | -,2929 | ,0458 | |
| SHORT  DIAMETER | Equal variances have been assumed | | ,104 | ,748 | -,3626 | | 686 | ,000 | -,2081 | | ,0574 | -,3208 | -,0954 | |
|  | Equal variances have not been assumed | |  |  | -,3626 | | 685,790 | ,000 | -,2081 | | ,0574 | -,3208 | -,0954 | |
| AREA | Equal variances have been assumed | | ,439 | ,508 | -2,408 | | 685 | ,013 | ,9460 | | ,3815 | -1,6950 | -,1969 | |
|  | Equal variances have not been assumed | |  |  | -2,408 | | 685,994 | ,013 | ,9460 | | ,3815 | -1,6950 | -,1969 | |
| MF-MIB | Equal variances have been assumed | | ,166 | ,694 | -,432 | | 686 | ,666 | -,0529 | | ,1225 | -,1876 | ,0260 | |
|  | Equal variances have not been assumed | |  |  | -,432 | | 685,997 | ,666 | -,0529 | | ,1225 | -,1876 | ,2934 | |
| MF-MSB | Equal variances have been assumed | | ,203 | ,652 | -1,280 | | 686 | ,201 | -,3265 | | ,2550 | -,8272 | ,1743 | |
|  | Equal variances have not been assumed | |  |  | -1,280 | | 685,821 | ,201 | -,3265 | | ,2550 | -,8272 | ,1743 | |

**3.- MF CHARACTERISTICS BY GENDER**

| **Group statistics** | | | | | |
| --- | --- | --- | --- | --- | --- |
|  | GENDER | N | MEAN | SD | Standart error of the mean |
| EMERGING ANGLE | male | 278 | 55,07 | 15,780 | ,946 |
|  | female | 410 | 52,35 | 15,916 | ,786 |
| LONG DIAMETER | male | 278 | 4,627 | 1,1685 | ,0701 |
|  | female | 410 | 4,314 | 1,0899 | ,0538 |
| SHORT DIAMETER | male | 278 | 3,104 | ,8407 | ,0504 |
|  | female | 410 | 2,798 | ,6725 | ,0332 |
| AREA | male | 278 | 11,712 | 5,5712 | ,3341 |
|  | female | 409 | 9,805 | 4,4614 | ,2206 |
| MF-MIB | male | 278 | 14,353 | 1,5984 | ,0959 |
|  | female | 410 | 13,006 | 1,3655 | ,0674 |
| MF-MSB | male | 278 | 11,976 | 3,0758 | ,1845 |
|  | female | 410 | 11,037 | 3,4705 | ,1714 |

| **Independent sample T-test** | | | | | | | | | | |
| --- | --- | --- | --- | --- | --- | --- | --- | --- | --- | --- |
|  | | Levene test for equality of variances | | T test for the equality of averages | | | | | | |
|  |  | F | Sig. | t | df | Sig. (bilateral) | Mean difference | Standart error of the difference | 95% confidence interval for the difference | |
|  |  |  |  |  |  |  |  |  | Inferior | Superior |
| EMERGING ANGLE | Equal variances have been assumed | ,023 | ,879 | 2,208 | 686 | ,028 | 2,721 | 1,232 | ,301 | 5,140 |
|  | Equal variances have not been assumed |  |  | 2,211 | 598,178 | ,027 | 2,721 | 1,230 | ,304 | 5,137 |
| LONG DIAMETER | Equal variances have been assumed | 2,063 | ,151 | 3,581 | 686 | ,000 | ,3122 | ,0872 | ,1410 | ,4834 |
|  | Equal variances have not been assumed |  |  | 3,533 | 566,662 | ,000 | ,3122 | ,0884 | ,1387 | ,4858 |
| SHORT DIAMETER | Equal variances have been assumed | 16,366 | ,000 | 5,279 | 686 | ,000 | ,3055 | ,0579 | ,1919 | ,4192 |
|  | Equal variances have not been assumed |  |  | 5,061 | 505,140 | ,000 | ,3055 | ,0604 | ,1869 | ,4242 |
| AREA | Equal variances have been assumed | 17,148 | ,000 | 4,967 | 685 | ,000 | 1,9072 | ,3840 | 1,1532 | 2,6612 |
|  | Equal variances have not been assumed |  |  | 4,763 | 505,854 | ,000 | 1,9072 | ,4004 | 1,1206 | 2,6938 |
| MF-MIB | Equal variances have been assumed | 7,026 | ,008 | 11,840 | 686 | ,000 | 1,3467 | ,1137 | 1,1233 | 1,5700 |
|  | Equal variances have not been assumed |  |  | 11,489 | 530,909 | ,000 | 1,3467 | ,1172 | 1,1164 | 1,5769 |
| MF-MSB | Equal variances have been assumed | 8,127 | ,004 | 3,645 | 686 | ,000 | ,9393 | ,2577 | ,4334 | 1,4453 |
|  | Equal variances have not been assumed |  |  | 3,730 | 639,108 | ,000 | ,9393 | ,2518 | ,4448 | 1,4338 |

**4.- MF CHARACTERISTICS BY AGE**

| **Group statistics** | | | | | |
| --- | --- | --- | --- | --- | --- |
|  | AGE GROUPS | N | MEAN | SD | Standart error of the mean |
| FM EMERGING ANGLE | < DE 50 | 336 | 49,44 | 13,645 | ,744 |
|  | > o = DE 50 | 352 | 57,28 | 16,953 | ,904 |
| FM LONG DIAMETER | < DE 50 | 336 | 4,494 | 1,1546 | ,0630 |
|  | > o = DE 50 | 352 | 4,390 | 1,1091 | ,0591 |
| FM SHORT DIAMETER | < DE 50 | 336 | 2,928 | ,7912 | ,0432 |
|  | > o = DE 50 | 352 | 2,916 | ,7289 | ,0389 |
| FM AREA | < DE 50 | 335 | 10,721 | 5,4188 | ,2961 |
|  | > o = DE 50 | 352 | 10,439 | 4,6222 | ,2464 |
| MF-MIB | < DE 50 | 336 | 13,440 | 1,7320 | ,0945 |
|  | > o = DE 50 | 352 | 13,655 | 1,4694 | ,0783 |
| MF-MSB | < DE 50 | 336 | 12,563 | 2,8434 | ,1551 |
|  | > o = DE 50 | 352 | 10,322 | 3,4267 | ,1826 |

| **Independent simple T-test** | | | | | | | | | | |
| --- | --- | --- | --- | --- | --- | --- | --- | --- | --- | --- |
|  | | Levene test for equality of variances | | T test for the equality of averages | | | | | | |
|  |  | F | Sig. | t | df | Sig. (bilateral) | Mean difference | Standart error of the difference | 95% confidence interval for the difference | |
|  |  |  |  |  |  |  |  |  | Inferior | Superior |
| EMERGING  ANGLE | Equal variances have been assumed | 14,420 | ,000 | -6,662 | 686 | ,000 | -7,838 | 1,177 | -10,148 | -5,528 |
|  | Equal variances have not been assumed |  |  | -6,695 | 667,141 | ,000 | -7,838 | 1,171 | -10,137 | -5,539 |
| LONG  DIAMETER | Equal variances have been assumed | ,235 | ,628 | 1,205 | 686 | ,229 | ,1040 | ,0863 | -,0655 | ,2734 |
|  | Equal variances have not been assumed |  |  | 1,204 | 680,877 | ,229 | ,1040 | ,0864 | -,0656 | ,2736 |
| SHORT  DIAMETER | Equal variances have been assumed | ,922 | ,337 | ,208 | 686 | ,835 | ,0121 | ,0580 | -,1018 | ,1259 |
|  | Equal variances have not been assumed |  |  | ,208 | 674,890 | ,836 | ,0121 | ,0581 | -,1020 | ,1261 |
| AREA | Equal variances have been assumed | 2,377 | ,124 | ,734 | 685 | ,463 | ,2815 | ,3837 | -,4718 | 1,0348 |
|  | Equal variances have not been assumed |  |  | ,731 | 656,961 | ,465 | ,2815 | ,3852 | -,4748 | 1,0378 |
| MF-MIB | Equal variances have been assumed | 11,475 | ,001 | -1,751 | 686 | ,080 | -,2141 | ,1223 | -,4541 | ,0260 |
|  | Equal variances have not been assumed |  |  | -1,744 | 657,323 | ,082 | -,2141 | ,1227 | -,4551 | ,0269 |
| MF-MSB | Equal variances have been assumed | 8,824 | ,003 | 9,311 | 686 | ,000 | 2,2406 | ,2407 | 1,7681 | 2,7131 |
|  | Equal variances have not been assumed |  |  | 9,351 | 673,067 | ,000 | 2,2406 | ,2396 | 1,7701 | 2,7111 |

**5.- MF CHARACTERISTICS BY DENTAL STATUS**

| **Descriptives** | | | | | | | | | |
| --- | --- | --- | --- | --- | --- | --- | --- | --- | --- |
|  | | N | Mean | SD | Standard error | 95% confidence interval for the mean | | Mínimum | Maximum |
|  |  |  |  |  |  | Inferior limit | Superior limit |  |  |
| EMERGING ANGLE | DENTATE | 614 | 52,07 | 14,686 | ,593 | 50,91 | 53,24 | 26 | 135 |
|  | PARCIALLY | 28 | 54,54 | 18,353 | 3,468 | 47,42 | 61,65 | 30 | 94 |
|  | EDENTULOUS | 46 | 71,20 | 19,333 | 2,850 | 65,45 | 76,94 | 32 | 104 |
|  | Total | 688 | 53,45 | 15,906 | ,606 | 52,26 | 54,64 | 26 | 135 |
| LONG  DIAMETER | DENTATE | 614 | 4,477 | 1,1248 | ,0454 | 4,388 | 4,566 | 1,9 | 8,7 |
|  | PARCIALLY | 28 | 4,421 | 1,0956 | ,2070 | 3,997 | 4,846 | 2,6 | 6,6 |
|  | EDENTULOUS | 46 | 3,970 | 1,1673 | ,1721 | 3,623 | 4,316 | 2,1 | 7,1 |
|  | Total | 688 | 4,441 | 1,1319 | ,0432 | 4,356 | 4,525 | 1,9 | 8,7 |
| SHORT  DIAMETER | DENTATE | 614 | 2,938 | ,7612 | ,0307 | 2,878 | 2,998 | 1,1 | 6,5 |
|  | PARCIALLY | 28 | 2,961 | ,6039 | ,1141 | 2,727 | 3,195 | 1,7 | 4,0 |
|  | EDENTULOUS | 46 | 2,676 | ,7922 | ,1168 | 2,441 | 2,911 | 1,2 | 4,3 |
|  | Total | 688 | 2,922 | ,7595 | ,0290 | 2,865 | 2,978 | 1,1 | 6,5 |
| AREA | DENTATE | 613 | 10,714 | 5,0730 | ,2049 | 10,312 | 11,116 | 1,7 | 40,3 |
|  | PARCIALLY | 28 | 10,571 | 4,1555 | ,7853 | 8,960 | 12,183 | 3,5 | 18,5 |
|  | EDENTULOUS | 46 | 8,750 | 4,5674 | ,6734 | 7,394 | 10,106 | 2,1 | 20,3 |
|  | Total | 687 | 10,577 | 5,0248 | ,1917 | 10,200 | 10,953 | 1,7 | 40,3 |
| MF-MIB | DENTATE | 614 | 13,528 | 1,5944 | ,0643 | 13,401 | 13,654 | 9,5 | 18,4 |
|  | PARCIALLY | 28 | 13,479 | 1,7933 | ,3389 | 12,783 | 14,174 | 11,1 | 17,3 |
|  | EDENTULOUS | 46 | 13,891 | 1,6320 | ,2406 | 13,407 | 14,376 | 10,4 | 18,8 |
|  | Total | 688 | 13,550 | 1,6054 | ,0612 | 13,430 | 13,670 | 9,5 | 18,8 |
| MF-MSB | DENTATE | 614 | 11,842 | 3,0243 | ,1221 | 11,603 | 12,082 | 3,6 | 21,3 |
|  | PARCIALLY | 28 | 9,689 | 3,1356 | ,5926 | 8,473 | 10,905 | 3,1 | 15,8 |
|  | EDENTULOUS | 46 | 6,778 | 3,7594 | ,5543 | 5,662 | 7,895 | ,0 | 14,6 |
|  | Total | 688 | 11,416 | 3,3463 | ,1276 | 11,166 | 11,667 | ,0 | 21,3 |

| **One factor ANOVA** | | | | | | |
| --- | --- | --- | --- | --- | --- | --- |
|  | | Sum of squares | df | Root mean square | F | Sig. |
| EMERGING ANGLE | Inter-groups | 15685,269 | 2 | 7842,635 | 33,973 | ,000 |
|  | Intra-groups | 158131,050 | 685 | 230,848 |  |  |
|  | Total | 173816,320 | 687 |  |  |  |
| LONG DIAMETER | Inter-groups | 11,017 | 2 | 5,509 | 4,341 | ,013 |
|  | Intra-groups | 869,241 | 685 | 1,269 |  |  |
|  | Total | 880,259 | 687 |  |  |  |
| SHORT DIAMETER | Inter-groups | 2,983 | 2 | 1,491 | 2,598 | ,075 |
|  | Intra-groups | 393,259 | 685 | ,574 |  |  |
|  | Total | 396,242 | 687 |  |  |  |
| AREA | Inter-groups | 165,037 | 2 | 82,518 | 3,290 | ,038 |
|  | Intra-groups | 17155,169 | 684 | 25,081 |  |  |
|  | Total | 17320,206 | 686 |  |  |  |
| MF-MIB | Inter-groups | 5,807 | 2 | 2,904 | 1,127 | ,325 |
|  | Intra-groups | 1764,913 | 685 | 2,577 |  |  |
|  | Total | 1770,720 | 687 |  |  |  |
| MF-MSB | Inter-groups | 1184,487 | 2 | 592,243 | 62,333 | ,000 |
|  | Intra-groups | 6508,364 | 685 | 9,501 |  |  |
|  | Total | 7692,851 | 687 |  |  |  |

**6. MF CHARACTERISTICS BY MF ANTEROPOSTERIOR POSITION**

|  | | N | Mean | SD | Standard error | 95% confidence interval for the mean | | Mínimum | Maximum |
| --- | --- | --- | --- | --- | --- | --- | --- | --- | --- |
|  |  |  |  |  |  | Inferior limit | Superior limit |  |  |
| EMERGING ANGLE | 1st MOLAR | 17 | 47,12 | 12,041 | 2,920 | 40,93 | 53,31 | 28 | 64 |
|  | 1st MOLAR-2nd PREMOLAR | 58 | 55,22 | 16,279 | 2,138 | 50,94 | 59,50 | 32 | 98 |
|  | 2nd PREMOLAR | 369 | 52,21 | 15,132 | ,788 | 50,66 | 53,76 | 26 | 135 |
|  | 2nd PREMOLAR-1st PREMOLAR | 162 | 52,73 | 14,034 | 1,103 | 50,56 | 54,91 | 28 | 97 |
|  | 1ºPREMOLAR | 32 | 52,66 | 15,774 | 2,788 | 46,97 | 58,34 | 31 | 92 |
|  | Total | 638 | 52,50 | 14,935 | ,591 | 51,34 | 53,66 | 26 | 135 |
| LONG DIAMETER | 1st MOLAR | 17 | 4,535 | 1,1921 | ,2891 | 3,922 | 5,148 | 2,7 | 7,3 |
|  | 1st MOLAR-2nd PREMOLAR | 58 | 4,774 | 1,1378 | ,1494 | 4,475 | 5,073 | 2,8 | 8,7 |
|  | 2nd PREMOLAR | 369 | 4,491 | 1,0342 | ,0538 | 4,385 | 4,597 | 2,2 | 8,3 |
|  | 2nd PREMOLAR-1st PREMOLAR | 162 | 4,412 | 1,2026 | ,0945 | 4,226 | 4,599 | 1,9 | 8,0 |
|  | 1ºPREMOLAR | 32 | 3,809 | 1,3973 | ,2470 | 3,306 | 4,313 | 2,3 | 7,1 |
|  | Total | 638 | 4,464 | 1,1232 | ,0445 | 4,377 | 4,551 | 1,9 | 8,7 |
| SHORT DIAMETER | 1st MOLAR | 17 | 3,165 | ,7770 | ,1884 | 2,765 | 3,564 | 1,6 | 4,9 |
|  | 1st MOLAR-2nd PREMOLAR | 58 | 3,241 | ,7854 | ,1031 | 3,035 | 3,448 | 2,0 | 5,3 |
|  | 2nd PREMOLAR | 369 | 2,955 | ,7228 | ,0376 | 2,881 | 3,029 | 1,6 | 6,5 |
|  | 2nd PREMOLAR-1st PREMOLAR | 162 | 2,854 | ,7661 | ,0602 | 2,735 | 2,973 | 1,1 | 5,4 |
|  | 1ºPREMOLAR | 32 | 2,453 | ,7915 | ,1399 | 2,168 | 2,738 | 1,2 | 4,2 |
|  | Total | 638 | 2,936 | ,7580 | ,0300 | 2,877 | 2,995 | 1,1 | 6,5 |
| AREA | 1st MOLAR | 17 | 11,647 | 5,0354 | 1,2213 | 9,058 | 14,236 | 4,1 | 21,9 |
|  | 1st MOLAR-2nd PREMOLAR | 58 | 12,466 | 5,4374 | ,7140 | 11,036 | 13,895 | 5,1 | 29,4 |
|  | 2nd PREMOLAR | 369 | 10,759 | 4,7693 | ,2483 | 10,271 | 11,247 | 3,1 | 40,3 |
|  | 2nd PREMOLAR-1st PREMOLAR | 162 | 10,364 | 5,1100 | ,4015 | 9,571 | 11,156 | 1,7 | 27,6 |
|  | 1ºPREMOLAR | 32 | 7,997 | 5,3698 | ,9493 | 6,061 | 9,933 | 2,7 | 22,5 |
|  | Total | 638 | 10,699 | 5,0131 | ,1985 | 10,309 | 11,089 | 1,7 | 40,3 |
| MF-MIB | 1st MOLAR | 17 | 14,465 | 2,0362 | ,4939 | 13,418 | 15,512 | 10,3 | 18,4 |
|  | 1st MOLAR-2nd PREMOLAR | 58 | 13,695 | 1,5422 | ,2025 | 13,289 | 14,100 | 10,3 | 17,7 |
|  | 2nd PREMOLAR | 369 | 13,552 | 1,6017 | ,0834 | 13,388 | 13,716 | 9,5 | 17,9 |
|  | 2nd PREMOLAR-1st PREMOLAR | 162 | 13,498 | 1,5480 | ,1216 | 13,258 | 13,738 | 10,1 | 17,6 |
|  | 1ºPREMOLAR | 32 | 13,672 | 1,7961 | ,3175 | 13,024 | 14,319 | 11,1 | 18,8 |
|  | Total | 638 | 13,582 | 1,6082 | ,0637 | 13,457 | 13,707 | 9,5 | 18,8 |
| MF-MIB | 1st MOLAR | 17 | 12,153 | 3,3649 | ,8161 | 10,423 | 13,883 | 5,6 | 19,5 |
|  | 1st MOLAR-2nd PREMOLAR | 58 | 11,231 | 3,6002 | ,4727 | 10,284 | 12,178 | 3,6 | 20,1 |
|  | 2nd PREMOLAR | 369 | 11,576 | 3,1193 | ,1624 | 11,257 | 11,896 | 3,1 | 21,3 |
|  | 2nd PREMOLAR-1st PREMOLAR | 162 | 12,178 | 2,5300 | ,1988 | 11,785 | 12,570 | 4,4 | 17,5 |
|  | 1ºPREMOLAR | 32 | 12,150 | 3,4895 | ,6169 | 10,892 | 13,408 | 4,8 | 17,9 |
|  | Total | 638 | 11,742 | 3,0621 | ,1212 | 11,504 | 11,980 | 3,1 | 21,3 |

| **One factor Anova** | | | | | | |
| --- | --- | --- | --- | --- | --- | --- |
|  | | Sum of squares | df | Root mean square | F | Sig. |
| EMERGING ANGLE | Inter-groups | 964,495 | 4 | 241,124 | 1,082 | ,365 |
|  | Intra-groups | 141117,003 | 633 | 222,934 |  |  |
|  | Total | 142081,498 | 637 |  |  |  |
| LONG DIAMETER | Inter-groups | 20,086 | 4 | 5,022 | 4,057 | ,003 |
|  | Intra-groups | 783,525 | 633 | 1,238 |  |  |
|  | Total | 803,611 | 637 |  |  |  |
| SHORT DIAMETER | Inter-groups | 14,974 | 4 | 3,743 | 6,751 | ,000 |
|  | Intra-groups | 351,014 | 633 | ,555 |  |  |
|  | Total | 365,988 | 637 |  |  |  |
| AREA | Inter-groups | 449,480 | 4 | 112,370 | 4,572 | ,001 |
|  | Intra-groups | 15559,317 | 633 | 24,580 |  |  |
|  | Total | 16008,797 | 637 |  |  |  |
| MF-MIB | Inter-groups | 15,713 | 4 | 3,928 | 1,524 | ,194 |
|  | Intra-groups | 1631,782 | 633 | 2,578 |  |  |
|  | Total | 1647,495 | 637 |  |  |  |
| MF-MSB | Inter-groups | 64,221 | 4 | 16,055 | 1,720 | ,144 |
|  | Intra-groups | 5908,551 | 633 | 9,334 |  |  |
|  | Total | 5972,773 | 637 |  |  |  |
