# Supplementary material for: Predictive factors of the dimensions and location of mental foramen using cone beam computed tomography
Source: PLoS One. 2017 Aug 17;12(8):e0179704. doi: 10.1371/journal.pone.0179704 (PMC5560523; doi:10.1371/journal.pone.0179704)
Supplement: S2 Dataset — (DOCX) [file pone.0179704.s002.docx]

**1. INTEROBSERVER VARIABILITY (INTRACLASS CORRELATION COEFICIENT)**

**MF EMERGING ANGLE-RIGHT**

**Intraclass correlation coefficient**

|  |  | Confidence interval of 95% | | Test F with true value 0 | | | | |
| --- | --- | --- | --- | --- | --- | --- | --- | --- |
|  | Intraclass correlation (a) | Lower limit | Upper limit | Value | gl1 | gl2 | Sig. | |
| Individual measures | ,699(b) | ,498 | ,829 | 5,644 | 39 | 39 | | ,000 |
| Average measures | ,823(c) | ,665 | ,906 | 5,644 | 39 | 39 | | ,000 |

Mixed effects model of two factors in which the effects of the people are random and the effects of the measures are fixed.

(a) Intraclass correlation coefficients of type C using a definition of coherence, the inter-measure variance is excluded from the variance of the denominator.

(b) The estimator is the same, whether or not the interaction effect is present.

(c) This estimate is calculated assuming that the interaction effect is not present, since otherwise it is not estimable.

**MF LONG DIAMETER-RIGHT**

**Intraclass correlation coefficient**

|  |  | Confidence interval of 95% | | Test F with true value 0 | | | | |
| --- | --- | --- | --- | --- | --- | --- | --- | --- |
|  | Intraclass correlation (b) | Lower limit | Upper limit | Value | gl1 | gl2 | Sig. | |
| Individual measures | ,623(a) | ,389 | ,781 | 4,303 | 39 | 39 | | ,000 |
| Average measures | ,768(c) | ,561 | ,877 | 4,303 | 39 | 39 | | ,000 |

Mixed effects model of two factors in which the effects of the people are random and the effects of the measures are fixed.

(a) Intraclass correlation coefficients of type C using a definition of coherence, the inter-measure variance is excluded from the variance of the denominator.

(b) The estimator is the same, whether or not the interaction effect is present.

(c) This estimate is calculated assuming that the interaction effect is not present, since otherwise it is not estimable.

**MF SHORT DIAMETER-RIGHT**

**Intraclass correlation coefficient**

|  |  | Confidence interval of 95% | | Test F with true value 0 | | | | | |
| --- | --- | --- | --- | --- | --- | --- | --- | --- | --- |
|  | Intraclass correlation (a) | Lower limit | Upper limit | Value | | gl1 | gl2 | Sig. | |
| Individual measures | ,766(b) | ,600 | ,869 | 7,557 | 39 | | 39 | | ,000 |
| Average measures | ,868(c) | ,750 | ,930 | 7,557 | 39 | | 39 | | ,000 |

Mixed effects model of two factors in which the effects of the people are random and the effects of the measures are fixed.

(a) Intraclass correlation coefficients of type C using a definition of coherence, the inter-measure variance is excluded from the variance of the denominator.

(b) The estimator is the same, whether or not the interaction effect is present.

(c) This estimate is calculated assuming that the interaction effect is not present, since otherwise it is not estimable.

**MF AREA -RIGHT**

| **Intraclass correlation coefficient** | | | | | | | |
| --- | --- | --- | --- | --- | --- | --- | --- |
|  | Intraclass correlation (b) | Confidence interval of 95% | | Test F with true value 0 | | | |
|  |  | Lower limit | Upper limit | Value | gl1 | gl2 | Sig. |
| Individual measures | ,782^a^ | ,624 | ,878 | 8,165 | 39 | 39 | ,000 |
| Average measures | ,878^c^ | ,768 | ,935 | 8,165 | 39 | 39 | ,000 |

Mixed effects model of two factors in which the effects of the people are random and the effects of the measures are fixed.

(a) Intraclass correlation coefficients of type C using a definition of coherence, the inter-measure variance is excluded from the variance of the denominator.

(b) The estimator is the same, whether or not the interaction effect is present.

(c) This estimate is calculated assuming that the interaction effect is not present, since otherwise it is not estimable.

**MF/MSB-RIGHT**

| **Intraclass correlation coefficient** | | | | | | | |
| --- | --- | --- | --- | --- | --- | --- | --- |
|  | Intraclass correlation (b) | Confidence interval of 95% | | Test F with true value 0 | | | |
|  |  | Lower limit | Upper limit | Value | gl1 | gl2 | Sig. |
| Individual measures | ,660^a^ | ,442 | ,805 | 4,882 | 39 | 39 | ,000 |
| Average measures | ,795^c^ | ,613 | ,892 | 4,882 | 39 | 39 | ,000 |

Mixed effects model of two factors in which the effects of the people are random and the effects of the measures are fixed.

(a) Intraclass correlation coefficients of type C using a definition of coherence, the inter-measure variance is excluded from the variance of the denominator.

(b) The estimator is the same, whether or not the interaction effect is present.

(c) This estimate is calculated assuming that the interaction effect is not present, since otherwise it is not estimable.

**MF/MIB-RIGHT**

**Intraclass correlation coefficient**

|  |  | Confidence interval of 95% | | Test F with true value 0 | | | | |
| --- | --- | --- | --- | --- | --- | --- | --- | --- |
|  | Intraclass correlation (a) | Lower limit | Upper limit | Value | gl1 | gl2 | Sig. | |
| Individual measures | ,815(b) | ,676 | ,898 | 9,793 | 39 | 39 | | ,000 |
| Average measures | ,898(c) | ,807 | ,946 | 9,793 | 39 | 39 | | ,000 |

Mixed effects model of two factors in which the effects of the people are random and the effects of the measures are fixed.

(a) Intraclass correlation coefficients of type C using a definition of coherence, the inter-measure variance is excluded from the variance of the denominator.

(b) The estimator is the same, whether or not the interaction effect is present.

(c) This estimate is calculated assuming that the interaction effect is not present, since otherwise it is not estimable.

**MF EMERGING ANGLE-LEFT**

**Intraclass correlation coefficient**

|  |  | Confidence interval of 95% | | Test F with true value 0 | | | | |
| --- | --- | --- | --- | --- | --- | --- | --- | --- |
|  | Intraclass correlation (a) | Lower limit | Upper limit | Value | gl1 | gl2 | Sig. | |
| Individual measures | ,853(b) | ,739 | ,919 | 12,581 | 39 | 39 | | ,000 |
| Average measures | ,921(c) | ,850 | ,958 | 12,581 | 39 | 39 | | ,000 |

Mixed effects model of two factors in which the effects of the people are random and the effects of the measures are fixed.

(a) Intraclass correlation coefficients of type C using a definition of coherence, the inter-measure variance is excluded from the variance of the denominator.

(b) The estimator is the same, whether or not the interaction effect is present.

(c) This estimate is calculated assuming that the interaction effect is not present, since otherwise it is not estimable.

**MF LONG DIAMETER-LEFT**

**Intraclass correlation coefficient**

|  |  | Confidence interval of 95% | | Test F with true value 0 | | | | |
| --- | --- | --- | --- | --- | --- | --- | --- | --- |
|  | Intraclass correlation (b) | Lower limit | Upper limit | Value | gl1 | gl2 | Sig. | |
| Individual measures | ,697(a) | ,496 | ,828 | 5,612 | 39 | 39 | | ,000 |
| Average measures | ,822(c) | ,663 | ,906 | 5,612 | 39 | 39 | | ,000 |

Mixed effects model of two factors in which the effects of the people are random and the effects of the measures are fixed.

(a) Intraclass correlation coefficients of type C using a definition of coherence, the inter-measure variance is excluded from the variance of the denominator.

(b) The estimator is the same, whether or not the interaction effect is present.

(c) This estimate is calculated assuming that the interaction effect is not present, since otherwise it is not estimable.

**MF SHORT DIAMETER-LEFT**

**Intraclass correlation coefficient**

|  |  | Confidence interval of 95% | | Test F with true value 0 | | | | |
| --- | --- | --- | --- | --- | --- | --- | --- | --- |
|  | Intraclass correlation (a) | Lower limit | Upper limit | Value | gl1 | gl2 | Sig. | |
| Individual measures | ,786(b) | ,631 | ,881 | 8,346 | 39 | 39 | | ,000 |
| Average measures | ,880(c) | ,773 | ,937 | 8,346 | 39 | 39 | | ,000 |

Mixed effects model of two factors in which the effects of the people are random and the effects of the measures are fixed.

(a) Intraclass correlation coefficients of type C using a definition of coherence, the inter-measure variance is excluded from the variance of the denominator.

(b) The estimator is the same, whether or not the interaction effect is present.

(c) This estimate is calculated assuming that the interaction effect is not present, since otherwise it is not estimable.

**MF AREA DIAMETER-LEFT**

**Intraclass correlation coefficient**

|  |  | Confidence interval of 95% | | Test F with true value 0 | | | | |
| --- | --- | --- | --- | --- | --- | --- | --- | --- |
|  | Intraclass correlation (b) | Lower limit | Upper limit | Value | gl1 | gl2 | Sig. | |
| Individual measures | ,765(a) | ,597 | ,868 | 7,499 | 39 | 39 | | ,000 |
| Average measures | ,867(c) | ,748 | ,929 | 7,499 | 39 | 39 | | ,000 |

Mixed effects model of two factors in which the effects of the people are random and the effects of the measures are fixed.

(a) Intraclass correlation coefficients of type C using a definition of coherence, the inter-measure variance is excluded from the variance of the denominator.

(b) The estimator is the same, whether or not the interaction effect is present.

(c) This estimate is calculated assuming that the interaction effect is not present, since otherwise it is not estimable.

**MF/MSB-LEFT**

**Intraclass correlation coefficient**

|  |  | Confidence interval of 95% | | Test F with true value 0 | | | | |
| --- | --- | --- | --- | --- | --- | --- | --- | --- |
|  | Intraclass correlation (b) | Lower limit | Upper limit | Value | gl1 | gl2 | Sig. | |
| Individual measures | ,714(a) | ,520 | ,838 | 5,982 | 39 | 39 | | ,000 |
| Average measures | ,833(c) | ,684 | ,912 | 5,982 | 39 | 39 | | ,000 |

Mixed effects model of two factors in which the effects of the people are random and the effects of the measures are fixed.

(a) Intraclass correlation coefficients of type C using a definition of coherence, the inter-measure variance is excluded from the variance of the denominator.

(b) The estimator is the same, whether or not the interaction effect is present.

(c) This estimate is calculated assuming that the interaction effect is not present, since otherwise it is not estimable.

**MF/MIB-LEFT**

**Intraclass correlation coefficient**

|  |  | Confidence interval of 95% | | Test F with true value 0 | | | | |
| --- | --- | --- | --- | --- | --- | --- | --- | --- |
|  | Intraclass correlation (b) | Lower limit | Upper limit | Value | gl1 | gl2 | Sig. | |
| Individual measures | ,820(a) | ,685 | ,901 | 10,122 | 39 | 39 | | ,000 |
| Average measures | ,901(c) | ,813 | ,948 | 10,122 | 39 | 39 | | ,000 |

Mixed effects model of two factors in which the effects of the people are random and the effects of the measures are fixed.

(a) Intraclass correlation coefficients of type C using a definition of coherence, the inter-measure variance is excluded from the variance of the denominator.

(b) The estimator is the same, whether or not the interaction effect is present.

(c) This estimate is calculated assuming that the interaction effect is not present, since otherwise it is not estimable.

**2. INTRAOBSERVER VARIABILITY (INTRACLASS CORRELATION COEFICIENT)**

**MF EMERGING ANGLE-RIGHT**

**Intraclass correlation coefficient**

|  |  | Confidence interval of 95% | | Test F with true value 0 | | | | |
| --- | --- | --- | --- | --- | --- | --- | --- | --- |
|  | Intraclass correlation (b) | Lower limit | Upper limit | Value | gl1 | gl2 | Sig. | |
| Individual measures | ,803(a) | ,658 | ,891 | 9,150 | 39 | 39 | | ,000 |
| Average measures | ,891(c) | ,793 | ,942 | 9,150 | 39 | 39 | | ,000 |

Mixed effects model of two factors in which the effects of the people are random and the effects of the measures are fixed.

(a) Intraclass correlation coefficients of type C using a definition of coherence, the inter-measure variance is excluded from the variance of the denominator.

(b) The estimator is the same, whether or not the interaction effect is present.

(c) This estimate is calculated assuming that the interaction effect is not present, since otherwise it is not estimable.

**MF LONG DIAMETER-RIGHT**

**Intraclass correlation coefficient**

|  |  | Confidence interval of 95% | | Test F with true value 0 | | | | |
| --- | --- | --- | --- | --- | --- | --- | --- | --- |
|  | Intraclass correlation (b) | Lower limit | Upper limit | Value | gl1 | gl2 | Sig. | |
| Individual measures | ,866(a) | ,761 | ,927 | 13,924 | 39 | 39 | | ,000 |
| Average measures | ,928(c) | ,864 | ,962 | 13,924 | 39 | 39 | | ,000 |

Mixed effects model of two factors in which the effects of the people are random and the effects of the measures are fixed.

(a) Intraclass correlation coefficients of type C using a definition of coherence, the inter-measure variance is excluded from the variance of the denominator.

(b) The estimator is the same, whether or not the interaction effect is present.

(c) This estimate is calculated assuming that the interaction effect is not present, since otherwise it is not estimable.

**MF SHORT DIAMETER-RIGHT**

**Intraclass correlation coefficient**

|  |  | Confidence interval of 95% | | Test F with true value 0 | | | | |
| --- | --- | --- | --- | --- | --- | --- | --- | --- |
|  | Intraclass correlation (b) | Lower limit | Upper limit | Value | gl1 | gl2 | Sig. | |
| Individual measures | ,910(a) | ,836 | ,951 | 21,126 | 39 | 39 | | ,000 |
| Average measures | ,953(c) | ,911 | ,975 | 21,126 | 39 | 39 | | ,000 |

Mixed effects model of two factors in which the effects of the people are random and the effects of the measures are fixed.

(a) Intraclass correlation coefficients of type C using a definition of coherence, the inter-measure variance is excluded from the variance of the denominator.

(b) The estimator is the same, whether or not the interaction effect is present.

(c) This estimate is calculated assuming that the interaction effect is not present, since otherwise it is not estimable.

**MF AREA -RIGHT**

**Intraclass correlation coefficient**

|  |  | Confidence interval of 95% | | Test F with true value 0 | | | | |
| --- | --- | --- | --- | --- | --- | --- | --- | --- |
|  | Intraclass correlation (b) | Lower limit | Upper limit | Value | gl1 | gl2 | Sig. | |
| Individual measures | ,907(a) | ,832 | ,950 | 20,615 | 39 | 39 | | ,000 |
| Average measures | ,951(c) | ,908 | ,974 | 20,615 | 39 | 39 | | ,000 |

Mixed effects model of two factors in which the effects of the people are random and the effects of the measures are fixed.

(a) Intraclass correlation coefficients of type C using a definition of coherence, the inter-measure variance is excluded from the variance of the denominator.

(b) The estimator is the same, whether or not the interaction effect is present.

(c) This estimate is calculated assuming that the interaction effect is not present, since otherwise it is not estimable.

**MF/MIB-RIGHT**

**Intraclass correlation coefficient**

|  |  | Confidence interval of 95% | | Test F with true value 0 | | | | |
| --- | --- | --- | --- | --- | --- | --- | --- | --- |
|  | Intraclass correlation (b) | Lower limit | Upper limit | Value | gl1 | gl2 | Sig. | |
| Individual measures | ,900(a) | ,819 | ,946 | 18,971 | 39 | 39 | | ,000 |
| Average measures | ,947(c) | ,900 | ,972 | 18,971 | 39 | 39 | | ,000 |

Mixed effects model of two factors in which the effects of the people are random and the effects of the measures are fixed.

(a) Intraclass correlation coefficients of type C using a definition of coherence, the inter-measure variance is excluded from the variance of the denominator.

(b) The estimator is the same, whether or not the interaction effect is present.

(c) This estimate is calculated assuming that the interaction effect is not present, since otherwise it is not estimable.

**MF/MSB-RIGHT**

**Intraclass correlation coefficient**

|  |  | Confidence interval of 95% | | Test F with true value 0 | | | | |
| --- | --- | --- | --- | --- | --- | --- | --- | --- |
|  | Intraclass correlation (b) | Lower limit | Upper limit | Value | gl1 | gl2 | Sig. | |
| Individual measures | ,899(a) | ,817 | ,945 | 18,825 | 39 | 39 | | ,000 |
| Average measures | ,947(c) | ,900 | ,972 | 18,825 | 39 | 39 | | ,000 |

Mixed effects model of two factors in which the effects of the people are random and the effects of the measures are fixed.

(a) Intraclass correlation coefficients of type C using a definition of coherence, the inter-measure variance is excluded from the variance of the denominator.

(b) The estimator is the same, whether or not the interaction effect is present.

(c) This estimate is calculated assuming that the interaction effect is not present, since otherwise it is not estimable.

**MF EMERGING ANGLE-LEFT**

**Intraclass correlation coefficient**

|  |  | Confidence interval of 95% | | Test F with true value 0 | | | | |
| --- | --- | --- | --- | --- | --- | --- | --- | --- |
|  | Intraclass correlation (b) | Lower limit | Upper limit | Value | gl1 | gl2 | Sig. | |
| Individual measures | ,729(b) | ,543 | ,847 | 6,380 | 39 | 39 | | ,000 |
| Average measures | ,843(c) | ,704 | ,917 | 6,380 | 39 | 39 | | ,000 |

Mixed effects model of two factors in which the effects of the people are random and the effects of the measures are fixed.

(a) Intraclass correlation coefficients of type C using a definition of coherence, the inter-measure variance is excluded from the variance of the denominator.

(b) The estimator is the same, whether or not the interaction effect is present.

(c) This estimate is calculated assuming that the interaction effect is not present, since otherwise it is not estimable.

**MF LONG DIAMETER-LEFT**

**Intraclass correlation coefficient**

|  |  | Confidence interval of 95% | | Test F with true value 0 | | | | |
| --- | --- | --- | --- | --- | --- | --- | --- | --- |
|  | Intraclass correlation (a) | Lower limit | Upper limit | Value | gl1 | gl2 | Sig. | |
| Individual measures | ,722(b) | ,532 | ,842 | 6,184 | 39 | 39 | | ,000 |
| Average measures | ,838(c) | ,694 | ,914 | 6,184 | 39 | 39 | | ,000 |

Mixed effects model of two factors in which the effects of the people are random and the effects of the measures are fixed.

(a) Intraclass correlation coefficients of type C using a definition of coherence, the inter-measure variance is excluded from the variance of the denominator.

(b) The estimator is the same, whether or not the interaction effect is present.

(c) This estimate is calculated assuming that the interaction effect is not present, since otherwise it is not estimable.

**MF SHORT DIAMETER-LEFT**

**Intraclass correlation coefficient**

|  |  | Confidence interval of 95% | | Test F with true value 0 | | | | |
| --- | --- | --- | --- | --- | --- | --- | --- | --- |
|  | Intraclass correlation (b) | Lower limit | Upper limit | Value | gl1 | gl2 | Sig. | |
| Individual measures | ,610(a) | ,372 | ,773 | 4,132 | 39 | 39 | | ,000 |
| Average measures | ,758(c) | ,542 | ,872 | 4,132 | 39 | 39 | | ,000 |

Mixed effects model of two factors in which the effects of the people are random and the effects of the measures are fixed.

(a) Intraclass correlation coefficients of type C using a definition of coherence, the inter-measure variance is excluded from the variance of the denominator.

(b) The estimator is the same, whether or not the interaction effect is present.

(c) This estimate is calculated assuming that the interaction effect is not present, since otherwise it is not estimable.

**MF AREA-LEFT**

**Intraclass correlation coefficient**

|  |  | Confidence interval of 95% | | Test F with true value 0 | | | | |
| --- | --- | --- | --- | --- | --- | --- | --- | --- |
|  | Intraclass correlation (b) | Lower limit | Upper limit | Value | gl1 | gl2 | Sig. | |
| Individual measures | ,740(a) | ,560 | ,854 | 6,700 | 39 | 39 | | ,000 |
| Average measures | ,851(c) | ,718 | ,921 | 6,700 | 39 | 39 | | ,000 |

Mixed effects model of two factors in which the effects of the people are random and the effects of the measures are fixed.

(a) Intraclass correlation coefficients of type C using a definition of coherence, the inter-measure variance is excluded from the variance of the denominator.

(b) The estimator is the same, whether or not the interaction effect is present.

(c) This estimate is calculated assuming that the interaction effect is not present, since otherwise it is not estimable.

**MF/MSB-LEFT**

**Intraclass correlation coefficient**

|  |  | Confidence interval of 95% | | Test F with true value 0 | | | | |
| --- | --- | --- | --- | --- | --- | --- | --- | --- |
|  | Intraclass correlation (b) | Lower limit | Upper limit | Value | gl1 | gl2 | Sig. | |
| Individual measures | ,758(a) | ,587 | ,864 | 7,256 | 39 | 39 | | ,000 |
| Average measures | ,862(c) | ,739 | ,927 | 7,256 | 39 | 39 | | ,000 |

Mixed effects model of two factors in which the effects of the people are random and the effects of the measures are fixed.

(a) Intraclass correlation coefficients of type C using a definition of coherence, the inter-measure variance is excluded from the variance of the denominator.

(b) The estimator is the same, whether or not the interaction effect is present.

(c) This estimate is calculated assuming that the interaction effect is not present, since otherwise it is not estimable.

**MF/MIB-LEFT**

**Intraclass correlation coefficient**

|  |  | Confidence interval of 95% | | Test F with true value 0 | | | | |
| --- | --- | --- | --- | --- | --- | --- | --- | --- |
|  | Intraclass correlation (b) | Lower limit | Upper limit | Value | gl1 | gl2 | Sig. | |
| Individual measures | ,752(a) | ,578 | ,861 | 7,077 | 39 | 39 | | ,000 |
| Average measures | ,859(c) | ,733 | ,925 | 7,077 | 39 | 39 | | ,000 |

Mixed effects model of two factors in which the effects of the people are random and the effects of the measures are fixed.

(a) Intraclass correlation coefficients of type C using a definition of coherence, the inter-measure variance is excluded from the variance of the denominator.

(b) The estimator is the same, whether or not the interaction effect is present.

(c) This estimate is calculated assuming that the interaction effect is not present, since otherwise it is not estimable.
