# Supplementary material for: Predictive factors of the dimensions and location of mental foramen using cone beam computed tomography
Source: PLoS One. 2017 Aug 17;12(8):e0179704. doi: 10.1371/journal.pone.0179704 (PMC5560523; doi:10.1371/journal.pone.0179704)
Supplement: S3 Dataset — (DOCX) [file pone.0179704.s003.docx]

**PREDICTION OF THE MENTAL FORAMEN DIMENSIONS (BINARY LOGISTIC REGRESSION)**

**Determining factors of the Long diameter of the MF**

| **VARIABLES** | | | **B** | **SE** | | **Wald** | **Exp (B)** | | | **[CI]** | |  |
| --- | --- | --- | --- | --- | --- | --- | --- | --- | --- | --- | --- | --- |
| Age | | | 0,037 | 0,007 | | 27,93 | 1,038 | | | [1,024-1,052] | |  |
| Gender (Female) | | | -0,530 | 0,2111 | | 6,31 | 0,58 | | | [0,389-0,890] | |  |
| Constant | | | 19,193 | 0,854 | | 0,000 |  | | | |  | |
| **χ^2^ (sig)** | **R2 Nagelkerke** | | | | **Hosmer & Lemenshow** | | | | **% Cases correctly classified** | | | |
| 5,825 (p=0,667) | | 0,180 | | 5,825 (p=0,667) | | | | 66,4% | | | | |

**SE: standar error; [CI]: confidence interval.**

**Determining factors of the Short diameter of the MF**

| **VARIABLES** | | | **B** | **SE** | | **Wald** | **Sig.** | | | **Exp (B) [CI]** |
| --- | --- | --- | --- | --- | --- | --- | --- | --- | --- | --- |
| Gender (Female) | | | -0,735 | 0,214 | | 11,807 | 0,001 | | | 0,479 [0,315-0,729] |
| Side (left) | | | 0,719 | 0,215 | | 11,181 | 0,001 | | | 2,051 [1,346-3,126] |
| FM position (1st molar) | | | 1,850 | 0,833 | | 4,935 | 0,026 | | | 6,358 [1,243-32,511] |
| FM position (1st molar-2nd premolar | | | 2,030 | 0,620 | | 10,734 | 0,001 | | | 7,618 [2,262 -25,654 ] |
| FM position (2nd premolar) | | | 1,039 | 0,500 | | 4,310 | 0,038 | | | 2,827 [1,060 -7,538 ] |
| FM position (2nd premolar-1st premolar) | | | 1,005 | 0,517 | | 3,777 | 0,052 | | | 2,73 [0,991- 7,532] |
| Presence of FMA | | | 0,901 | 0,434 | | 4,305 | 0,038 | | | 2,462 [1,051- 5,765] |
| Constant | | | -1,651 | -0,650 | | 6,448 | 0,011 | | | 0,192 |
| **χ^2^ (sig)** | **R2 Nagelkerke** | | | | **Hosmer & Lemenshow** | | | | **% Cases correctly classified** | |
| 46,764 (p=0,000) | | 0,146 | | 2,410 (p=0,934) | | | | 64,5% | | |

**SE: standar error; [CI]: confidence interval.**

**Determining factors of the Area of the MF**

| **VARIABLES** | | | **B** | **SE** | | **Wald** | **Sig.** | | | **Exp (B) [CI]** |
| --- | --- | --- | --- | --- | --- | --- | --- | --- | --- | --- |
| Gender (Female) | | | -0,608 | 0,201 | | 9,135 | 0,003 | | | 0,544 [1,00-1,03] |
| Side (left) | | | 0,558 | 0,199 | | 7,873 | 0,005 | | | 1,744 [0,09-0,22] |
| Presence of FMA | | | 0,852 | 0,410 | | 4,314 | 0,038 | | | 2,345 [0,95-0,98] |
| Constant | | | -0,635 | 0,426 | | 2,223 | 0,136 | | | 0,530 |
| **χ^2^ (sig)** | **R2 Nagelkerke** | | | | **Hosmer & Lemenshow** | | | | **% Cases correctly classified** | |
| 21,793 (p=0,000) | | 0,066 | | 0,517 (p=0,972) | | | | 60% | | |

**SE: standar error; [CI]: confidence interval.**
