# Supplementary material for: Predictive factors of the dimensions and location of mental foramen using cone beam computed tomography
Source: PLoS One. 2017 Aug 17;12(8):e0179704. doi: 10.1371/journal.pone.0179704 (PMC5560523; doi:10.1371/journal.pone.0179704)
Supplement: S4 Dataset — (DOCX) [file pone.0179704.s004.docx]

**PREDICTION OF THE MENTAL FORAMEN LOCATION (BINARY LOGISTIC REGRESSION)**

**Determining factors of MF-MSB distance**

| **VARIABLES** | | | **B** | **SE** | | **Wald** | **Sig.** | | | **Exp (B) [CI]** |
| --- | --- | --- | --- | --- | --- | --- | --- | --- | --- | --- |
| Age | | | -0,054 | 0,007 | | 53,12 | 0,001 | | | 0,95 [0,93-0,96] |
| Gender (Female) | | | -0,94 | 0,22 | | 17,55 | 0,001 | | | 0,39 [0,25-0,61] |
| Dental stauts (Dentate) | | | 2,27 | 0,63 | | 12,99 | 0,001 | | | 9,67 [2,82-3,18] |
| Dental status (Partially dentate) | | | 1,10 | 0,85 | | 1,67 | 0,19 | | | 3,01 [0,57-15,96] |
| Constant | | | 1,06 | 0,76 | | 1,965 | 0,16 | | | 2,88 |
| **χ^2^ (sig)** | **R2 Nagelkerke** | | | | **Hosmer & Lemenshow** | | | | **% Cases correctly classified** | |
| 123,78 (p<0,001) | | 0,30 | | 11,83 (p=0,15) | | | | 72,1% | | |

**SE: standar error; [CI]: confidence interval.**

**Determining factors of MF-MIB distance**

| **VARIABLES** | | | **B** | **SE** | | **Wald** | **Sig.** | | | **Exp (B) [CI]** |
| --- | --- | --- | --- | --- | --- | --- | --- | --- | --- | --- |
| Age | | | -0,018 | 0,007 | | 6,67 | 0,01 | | | 1,01 [1,00-1,03] |
| Gender (Female) | | | -1,94 | 0,217 | | 79,69 | 0,001 | | | 0,14 [0,09-0,22] |
| Emerging angle | | | -0,03 | 0,007 | | 17,05 | 0,001 | | | 0,97 [0,95-0,98] |
| Constant | | | 1,86 | 0,473 | | 15,58 | 0,001 | | | 6,46 |
| **χ^2^ (sig)** | **R2 Nagelkerke** | | | | **Hosmer & Lemenshow** | | | | **% Cases correctly classified** | |
| 110,68 (p=0,001) | | 0,28 | | 11,59 (p=0,19) | | | | 71,2% | | |

**SE: standar error; [CI]: confidence interval.**

**Determining factors of MV distance**

| **VARIABLES** | | | **B** | **SE** | | **Wald** | **Sig.** | | | **Exp (B) [CI]** |
| --- | --- | --- | --- | --- | --- | --- | --- | --- | --- | --- |
| Age | | | -0,041 | 0,008 | | 27,741 | 0,000 | | | 0,959 [0,945-0,974] |
| Gender (Female) | | | -1,964 | 0,250 | | 61,860 | 0,000 | | | 0,140 [0,086-0,229] |
| Dental status (dentate) | | | 1,597 | 0,492 | | 10,520 | 0,001 | | | 4,938 [1,881-12,963] |
| Dental status (partially dentate) | | | 1,035 | 0,744 | | 1,935 | 0,164 | | | 2,814 [0,655-12,095] |
| Emerging angle | | | -0,031 | 0,008 | | 15,892 | 0,000 | | | 0,970 [0,955-0,984] |
| Constant | | | 3,422 | 0,782 | | 19,127 | 0,000 | | | 30,633 |
| **χ^2^ (sig)** | **R2 Nagelkerke** | | | | **Hosmer & Lemenshow** | | | | **% Cases correctly classified** | |
| 151,647 (p=0,000) | | 0,369 | | 13,879 (p=0,085) | | | | 76,3% | | |

**SE: standar error; [CI]: confidence interval.**

**Determining factors of MF-MSB/MV Ratio**

| **VARIABLES** | | | **B** | **SE** | | **Wald** | **Sig.** | | | **Exp (B) [CI]** |
| --- | --- | --- | --- | --- | --- | --- | --- | --- | --- | --- |
| Gender | | | -0,505 | 0,195 | | 60701 | 0,010 | | | 0,604 [0,412-0,885] |
| Presence of FMA | | | -0,804 | 0,396 | | 4,27 | 0,042 | | | 0,233 [1,029-4,849] |
| Constant | | | -0,320 | 0,391 | | 0,669 | 0,413 | | | 0,723 |
| **χ^2^ (sig)** | **R2 Nagelkerke** | | | | **Hosmer & Lemenshow** | | | | **% casos correctamente clasificados** | |
| 10,431 (p=0,05) | | 0,031 | | 0,03 (p=0,957) | | | | 55,7% | | |

**SE: standar error; [CI]: confidence interval.**
